# Age-related GSK3**β** overexpression drives podocyte senescence and glomerular aging

**DOI:** 10.1172/JCI141848

**Published:** 2022-02-15

**Authors:** Yudong Fang, Bohan Chen, Zhangsuo Liu, Athena Y. Gong, William T. Gunning, Yan Ge, Deepak Malhotra, Amira F. Gohara, Lance D. Dworkin, Rujun Gong

**Affiliations:** 1 Division of Nephrology, Department of Medicine and; 2 Center for Hypertension and Precision Medicine, University of Toledo College of Medicine, Toledo, Ohio, USA.; 3 Department of Nephrology, the First Affiliated Hospital of Zhengzhou University, Zhengzhou, China.; 4 Division of Kidney Disease and Hypertension, Rhode Island Hospital and The Warren Alpert Medical School of Brown University, Providence, Rhode Island, USA.; 5 Department of Pathology and; 6 Department of Physiology and Pharmacology, University of Toledo College of Medicine, Toledo, Ohio, USA.

**Keywords:** Aging, Nephrology, Bipolar disorder, Cellular senescence, Cytoskeleton

## Abstract

As life expectancy continues to increase, clinicians are challenged by age-related renal impairment that involves podocyte senescence and glomerulosclerosis. There is now compelling evidence that lithium has a potent antiaging activity that ameliorates brain aging and increases longevity in *Drosophila* and *Caenorhabditis elegans*. As the major molecular target of lithium action and a multitasking protein kinase recently implicated in a variety of renal diseases, glycogen synthase kinase 3**β** (GSK3**β**) is overexpressed and hyperactive with age in glomerular podocytes, correlating with functional and histological signs of kidney aging. Moreover, podocyte-specific ablation of GSK3**β** substantially attenuated podocyte senescence and glomerular aging in mice. Mechanistically, key mediators of senescence signaling, such as p16^INK4A^ and p53, contain high numbers of GSK3**β** consensus motifs, physically interact with GSK3**β**, and act as its putative substrates. In addition, therapeutic targeting of GSK3**β** by microdose lithium later in life reduced senescence signaling and delayed kidney aging in mice. Furthermore, in psychiatric patients, lithium carbonate therapy inhibited GSK3**β** activity and mitigated senescence signaling in urinary exfoliated podocytes and was associated with preservation of kidney function. Thus, GSK3**β** appears to play a key role in podocyte senescence by modulating senescence signaling and may be an actionable senostatic target to delay kidney aging.

## Introduction

Thanks to improvements in living conditions, public health interventions, and progress in medical care, human life expectancy has increased significantly in the past several decades (1). One consequence of these changes is the rapid aging of the world population. Human aging is associated with molecular, structural, and functional changes in a variety of organ systems, including the kidney. With age, many individuals experience a progressive decline in kidney function as well as macroscopic and microscopic histologic alterations, with the key feature being glomerular aging marked by global glomerulosclerosis (2, 3). Glomerular aging involves senescence of various glomerular cells, in particular the terminally differentiated podocytes, which are a critical constituent of the glomerular filtration barrier (3, 4). In addition, a characteristic senescence-associated secretory phenotype (SASP) is a pathognomonic feature of senescence in myriad tissues, including the glomeruli (1, 3–5). Senescent glomerular cells like podocytes may affect nearby cells in the glomeruli via profibrogenic SASP paracrine signaling and cause glomerulosclerosis (5). Age-related changes in the kidney may be accentuated by common comorbidities, such as hypertension (1, 5) and diabetes mellitus (6), or by preexisting kidney diseases (5). As life expectancy continues to improve worldwide, clinicians will be increasingly challenged by kidney aging, which predisposes to a high burden of kidney diseases and systemic comorbidities (5). There exists an immense unmet need to understand the molecular mechanism underlying renal cell senescence and to develop a pragmatic actionable target for retarding the process of kidney aging.

Research on the biology of aging has identified a number of chemical agents that possess antiaging activity. Among these, lithium, as a simple and common alkali metal ion, has been shown to have potent aging-delaying or longevity/lifespan-increasing effects and hence attracted much interest (7, 8). In *Caenorhabditis* *elegans*, lithium administration in clinically relevant concentrations increased survival by nearly 50% during normal aging (7, 9). More recently, in *Drosophila*, lithium, administered either throughout adulthood or only later in life, substantially promoted longevity and health span (8). In fact, for centuries, lithium has been regarded as a wonder drug that is capable of exerting miraculous effects on the body (10). It has been noted for a long time that low-dose lithium may promote longevity in humans. In support of this, large-scale surveys revealed that lithium concentrations in drinking water were inversely correlated with all-cause mortality in Japan (7) and in the United States (11). In addition, lithium ameliorates certain neurodegenerative diseases, such as Alzheimer’s disease, by suppressing the formation of β-amyloid plaques by suppressing β-amyloid pathology (12, 13), a hallmark and presumed causative factor of brain aging. Furthermore, leukocyte telomeres in humans taking lithium over long periods were approximately 35% longer than non–lithium-treated controls (14, 15), indicating protection against telomere shortening, a key mechanism implicated in aging (16). As a potent mood stabilizer, lithium has been commonly and safely used for over 50 years as a US FDA–approved first-line therapy for affective disorders (10). Of note, the usual psychiatric doses of lithium are relatively high and sometimes, though uncommonly, associated with lithium toxicity to peripheral organs, including the kidney (17, 18). However, despite occasional reports of lithium nephrotoxicity in psychiatric patients, the actual effect of microdose lithium on renal pathobiology and kidney aging has been barely studied.

Biochemically, lithium affects numerous cell signaling pathways. Among these, glycogen synthase kinase 3 (GSK3) has been shown to be the major molecular target of lithium action (19, 20). GSK3 is a highly conserved, ubiquitously expressed, and constitutively active serine/threonine protein kinase, originally characterized as a key transducer of the insulin signaling cascade that governs glycogenesis (20). Interest in GSK3 increased greatly with the realization that it also plays a pivotal role in a variety of other key signaling pathways involved in embryogenesis, tissue injury, repair, and regeneration (20). GSK3 exists as 2 isoforms: GSK3α and GSK3β (20). The 2 isoforms are differentially expressed in a tissue-dependent pattern. In renal glomeruli, the β rather than the α isoform of GSK3 is predominantly expressed and particularly enriched in podocytes (21). Combined knockout (KO) of both GSK3α and GSK3β specifically in glomerular podocytes in embryonic or adult mice caused severe podocyte injury, glomerulosclerosis, and heavy proteinuria (22). In contrast, podocyte-specific KO of only GSK3β in mice either at the embryonic stage (22) or during young adulthood (21, 22) resulted in no discernible phenotype. There is also burgeoning evidence suggesting that therapeutic targeting of GSK3β is likely renoprotective. To this end, GSK3β has been shown to play a key role in regulating injury of various kidney cells, like podocytes (21), and is centrally involved in pathogenesis of diverse kidney diseases, such as glomerular disease (23), acute kidney injury (AKI) (24, 25), diabetic nephropathy (26), and chronic kidney disease (CKD) (27, 28). However, the role of GSK3β in renal cell senescence and kidney aging remains unknown. This study aims to define this role and to test the effect of genetic or lithium targeting of GSK3β on kidney aging.

## Results

### Kidney aging is associated with GSK3β overexpression in glomerular podocytes.

Age-related changes in the kidney have been described previously (1–3). In non-neoplastic nephrectomy specimens procured from patients of varying ages (Supplemental Figure 1, A–J; supplemental material available online with this article; https://doi.org/10.1172/JCI141848DS1), hallmarks of kidney aging were evidently noted in the aged group, including nephrosclerosis, glomerulomegaly, glomerular basement membrane (GBM) thickening, mesangial expansion, global glomerulosclerosis, focal tubular atrophy, interstitial fibrosis, and accumulation of extracellular matrix in both glomeruli and interstitium, as shown by periodic acid–Schiff (PAS) and Masson’s trichrome staining (Supplemental Figure 1, A and B). Semiquantitative morphometric analysis revealed consistent, age-related increases in the percentage of globally sclerotic glomeruli and the extent of interstitial fibrosis (Supplemental Figure 1, E and F). Signs of age-related podocyte degeneration (2, 4) were also noted, including reduced expression of podocyte marker proteins podocin and Wilms’ tumor 1 (WT-1) on fluorescent immunohistochemical staining (Supplemental Figure 1D), indicative of podocyte loss, and ultrastructural lesions (Supplemental Figure 1C), such as variable effacement of podocyte foot processes, podocytopenia, and cytoplasmic absorption droplets in podocytes. Morphometric analysis (Supplemental Figure 1, G and H) confirmed the histologic observations and demonstrated an age-dependent podocyte foot process broadening and depletion of WT-1–positive podocytes. The histological changes were associated with a modest but statistically significant decline in kidney function as evaluated by estimated glomerular filtration rate (eGFR) (Supplemental Figure 1I). Moreover, linear regression analysis (Supplemental Figure 1J) revealed a positive correlation between the average number of WT-1–positive podocytes per glomerular cross section and eGFR, consistent with a possible causative role of podocyte senescence in age-related decline in kidney function.

**Figure 1:**
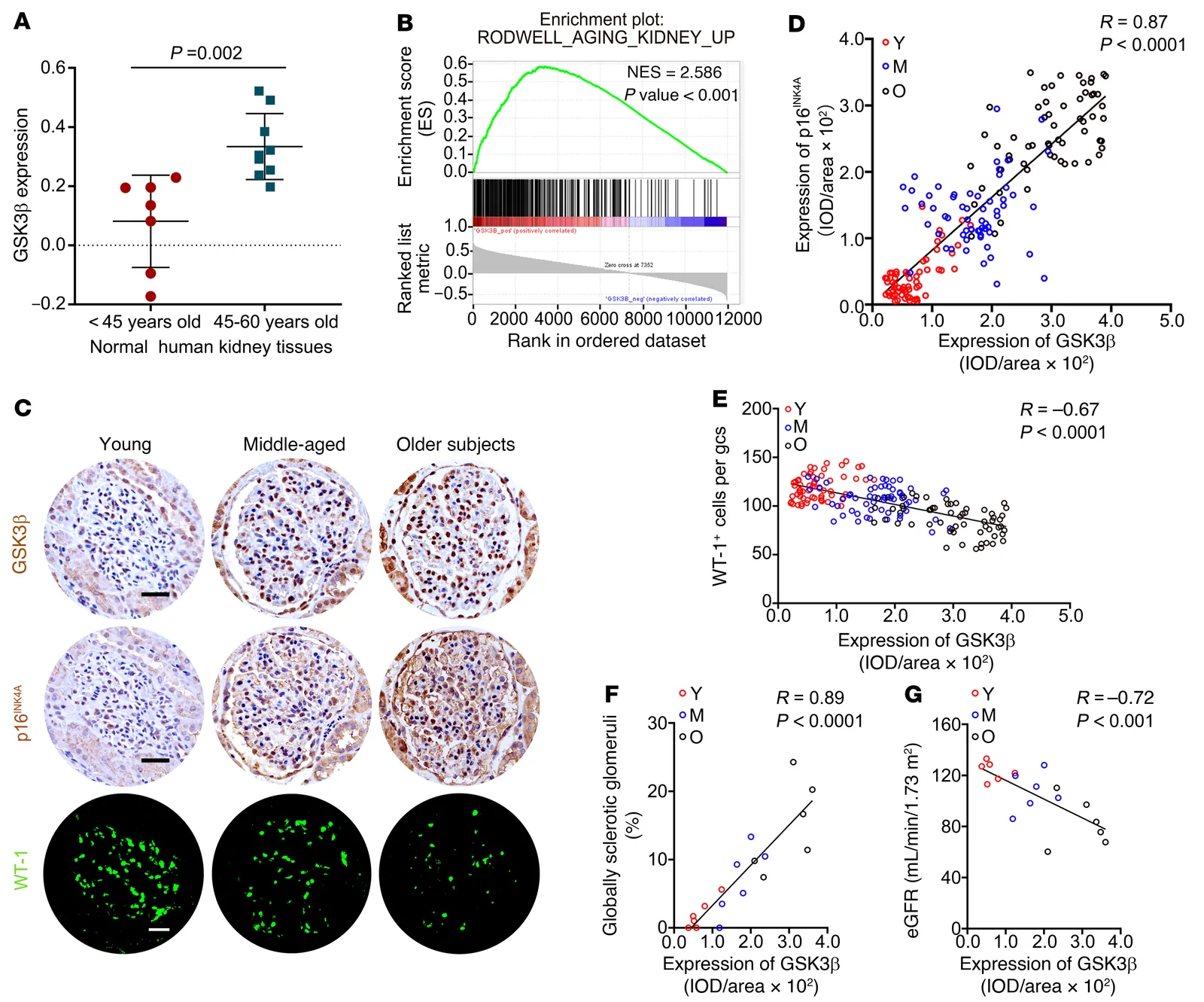
GSK3β expression in glomeruli increases with age and is mainly localized to glomerular podocytes. (**A**) Post hoc analysis of the renal cortical transcriptome was conducted based on the Nephroseq data set derived from the Rodwell Aging Kidney study, with exclusion of subjects with abnormal serum creatinine levels or blood pressure, or other comorbid conditions. The mRNA expression levels of GSK3β, expressed as log_2_ median-centered intensity, are shown for subjects aged 45 to 60 years (*n =* 9) versus younger subjects (*n =* 7). *P* value is shown. (**B**) Gene set enrichment analysis of glomerular transcriptome derived from the Ju CKD Glom data set demonstrated that the expert-curated kidney-aging-related gene set RODWELL_AGING_KIDNEY_UP is enriched in high GSK3β expression phenotype. Normalized enrichment score (NES) and nominal *P* values are shown. (**C**) Non-neoplastic nephrectomy specimens were procured from patients of varying ages (young or Y, <30 years old; middle-aged or M, 30 to 59 years old; older subjects or O, 60 to 79 years old) as elaborated in Supplemental Figure 1. Consecutive kidney sections were subjected to immunohistochemical staining for GSK3β and p16^INK4A^, along with immunofluorescent staining for WT-1. Scale bars: 20 μm. (**D** and **E**) Linear regression analyses of the relative glomerular staining intensity of GSK3β and (**D**) that of p16^INK4A^ or (**E**) the number of WT-1–positive podocytes per glomerular cross section (gcs) per subject (*n* = 6 subjects per group, 60 glomeruli analyzed per group with 10 per subject). IOD, integrated optical density. (**F** and **G**) Linear regression analyses show that the average relative glomerular staining intensity of GSK3β (**F**) positively correlated with the percentage of global glomerulosclerosis and (**G**) inversely correlated with estimated glomerular filtration rate (eGFR) (*n =* 6). Spearman’s correlation coefficient (*R*) and *P* value are shown. Panel **A** was analyzed with 2-tailed, unpaired Student’s *t* test. Panels **D**–**G** were statistically analyzed by linear regression.

Recognizing the potential antiaging effects of lithium (7, 8, 10–12, 14), we examined the expression of GSK3β, the molecular target of lithium action, in the kidney during aging. A post hoc analysis of the renal cortical transcriptome was conducted based on the Nephroseq data sets derived from the Rodwell Aging Kidney study (29) of normal kidney tissues procured at nephrectomy for either removal of a tumor or transplantation. As shown in Figure 1A, renal cortical expression of GSK3β in subjects aged 45 to 60 years was significantly higher than that in younger subjects. Moreover, as determined by gene set enrichment analysis (GSEA) (Figure 1B), the expert-curated kidney-aging-related gene set RODWELL_AGING_KIDNEY_ UP exhibited significant enrichment in high expression of GSK3β as compared with low expression of GSK3β in normal and diseased glomeruli based on the Ju CKD Glom data set (30). To verify the bioinformatics data, consecutive sections of non-neoplastic nephrectomy specimens procured from patients of different ages were subjected to immunostaining for GSK3β, the senescence marker p16^INK4A^ (31), and the podocyte marker WT-1. As shown in Figure 1C, glomerular expression of GSK3β was lowest in the kidneys of young subjects. This expression progressively increased with age in both cytoplasm and some nuclei, and was mostly localized to the periphery of glomerular tufts in cells that also stained positive for both WT-1 and p16^INK4A^, hence suggesting the coexistence of senescence and GSK3β overexpression in glomerular podocytes. Furthermore, computerized morphometric analysis confirmed that glomerular expression levels of GSK3β correlated positively with those of p16^INK4A^ (Figure 1D) but negatively with the number of WT-1–positive podocytes per glomerular cross section (Figure 1E). Moreover, glomerular expression levels of GSK3β also correlated positively with the severity of glomerulosclerosis (Figure 1F) but negatively with eGFR (Figure 1G). Collectively, these findings suggest that glomerular overexpression of GSK3β is associated with kidney aging, podocytopenia, and decline in kidney function during the aging process in humans.

### Murine models of normal aging also demonstrate an association between podocyte senescence and increased expression of GSK3β.

To validate the above findings, we examined a relevant, well-characterized, and extensively researched murine model of human aging (32) in wild-type (WT) mice (Supplemental Figure 2A). Mice exhibited a modest but consistent and significant decline in kidney function that associated with age, as assessed by serum creatinine levels (Supplemental Figure 2B), and proteinuria, measured by urinary protein electrophoresis and by urinary albumin-to-creatinine ratios (Supplemental Figure 2C), suggesting a progressive impairment of the glomerular filtration barrier function. Moreover, PAS staining revealed that global glomerulosclerosis was increased in older mice (Supplemental Figure 2D), along with other features of kidney aging such as tubular atrophy and interstitial fibrosis. In addition, podocyte senescence and degeneration were more pronounced in older mice, marked by variable broadening of podocyte foot processes (Supplemental Figure 2, E and F), podocytopenia, cytoplasmic inclusions in podocytes, GBM thickening on electron microscopy, and distribution in the periphery of glomerular tufts of staining for the acidic senescence-associated β‑galactosidase (SA-β-gal) activity (Supplemental Figure 2, E and G), one of the most reliable biomarkers of cellular senescence (31). In concert with podocyte senescence, the average number of WT-1–positive podocytes in glomerular cross sections was diminished in aged mice (Supplemental Figure 2, H and I), indicative of podocytopenia. This was associated with reduced glomerular expression of the homeostatic podocyte marker podocin and increased accumulation of the extracellular matrix protein fibronectin, as shown by fluorescent immunohistochemical staining (Supplemental Figure 2H) and by immunoblot analysis of isolated glomeruli (Supplemental Figure 2J), consistent with glomerular sclerosis.

**Figure 2:**
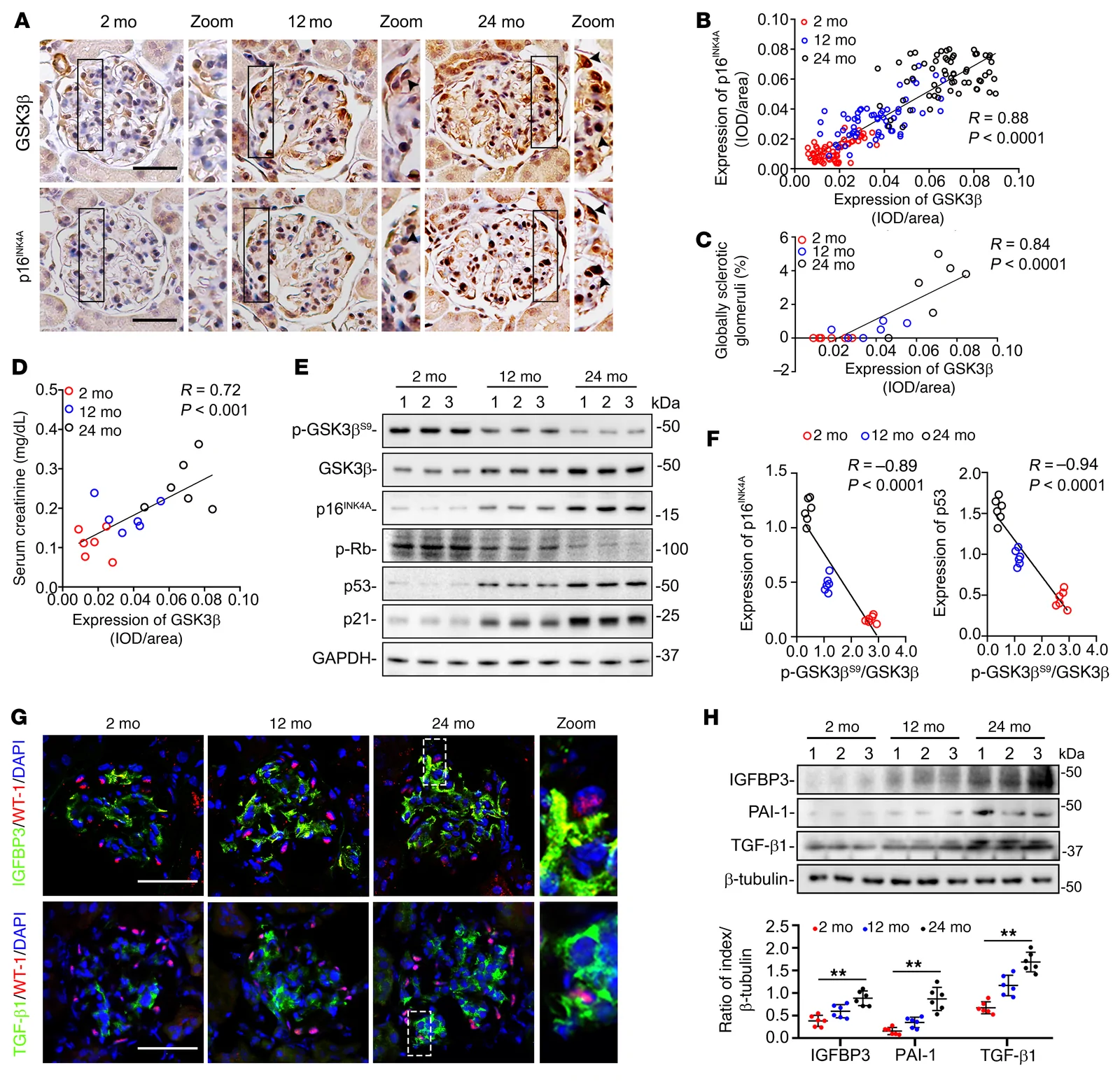
GSK3β is overexpressed and hyperactive in glomerular podocytes during the aging process in mice, and is associated with podocyte senescence, senescence-associated secretory phenotypes (SASPs), and kidney aging. (**A**) Mice were treated as elaborated in Supplemental Figure 2. Consecutive kidney sections collected at 2, 12, or 24 months of age (mo) were subjected to peroxidase immunohistochemical staining. Zoomed-in views of boxed areas show positive podocyte staining, as indicated by arrowheads. Scale bars: 20 μm and 4 μm (zoomed-in images). (**B**) Linear regression analysis reveals a significant correlation between the relative glomerular staining intensity of GSK3β and that of p16^INK4A^, as estimated by computerized morphometric analysis (*n* = 6 mice, 60 glomeruli were analyzed per group with 10 per mouse). IOD, integrated optical density. (**C** and **D**) Linear regression analysis reveals significant correlations between the average relative glomerular staining intensity of GSK3β and (**C**) the percentage of global glomerulosclerosis or (**D**) serum creatinine levels (*n =* 6). (**E**) Representative immunoblot analysis of isolated glomeruli for indicated proteins. GAPDH served as a loading control. (**F**) Linear regression analysis showed an inverse correlation between the relative p-GSK3β^S9^/GSK3β ratios and the relative expression levels of p16^INK4A^ or p53 in glomeruli based on densitometric analysis of immunoblots (*n =* 6). (**G**) Kidney tissues were subjected to fluorescent immunohistochemical staining. Zoomed-in views of boxed areas show positive staining for SASP factors in WT-1^+^ podocytes. Scale bars: 30 μm (left 3 columns) and 3 μm (zoomed-in images). (**H**) Representative immunoblot of isolated glomeruli analyzed for SASP factors. Densitometric analyses of the expression levels of diverse SASP factors in glomeruli, presented as relative levels normalized to β-tubulin based on immunoblot analysis. **P < 0.01 among different age groups (*n =* 6). Data are expressed as mean ± SD. Spearman’s correlation coefficient (*R*) and *P* value are shown in panels **B**–**D** and **F**. Panel **H** was analyzed by 1-way ANOVA.

To determine whether the pattern of GSK3β expression in the kidney changed with age, consecutive mouse kidney sections were examined by peroxidase immunostaining for GSK3β (Figure 2A) and for the senescence marker p16^INK4A^. In murine glomeruli, GSK3β expression was mostly probed in glomerular cells positive for podocin, as shown by dual-color fluorescent immunohistochemical staining (Supplemental Figure 3), suggesting a podocyte-enriched expression pattern. This expression was increased with age (Figure 2A), in parallel with enhanced glomerular and podocyte expression of p16^INK4A^ (Figure 2A). Linear regression analysis revealed a positive correlation between GSK3β and p16^INK4A^ expression in glomeruli, as determined by computerized morphometric analysis (Figure 2B). Moreover, glomerular expression levels of GSK3β correlated with the severity of glomerulosclerosis (Figure 2C) and with serum creatinine (Figure 2D). Immunoblot analysis of isolated glomeruli confirmed the morphologic findings and demonstrated progressively increased expression of GSK3β with age, along with augmented expression of the senescence signaling mediators (31) p16^INK4A^, p53, and p21, and repressed expression of phosphorylated Rb (p-Rb) (Figure 2E). Inhibitory phosphorylation of GSK3β^S9^ was also reduced, consistent with GSK3β hyperactivity. Linear regression analysis revealed a negative correlation between p-GSK3β^S9^/GSK3β ratios, as estimated by densitometric analysis of immunoblots, and the expression of p16^INK4A^ or p53 (Figure 2F), suggesting an association between GSK3β hyperactivity and senescence in glomerular cells. Accompanying the age-dependent glomerular overexpression of GSK3β, the expression of a subset of SASP factors implicated in fibrogenesis, TGF-β1, IGFBP3, and PAI-1 (1, 4, 5), also increased in glomeruli, as shown by immunohistochemical staining (Figure 2G) or by immunoblot analysis of isolated glomeruli (Figure 2H).

### Podocyte-specific ablation of GSK3β attenuates kidney aging in mice.

**Figure 3:**
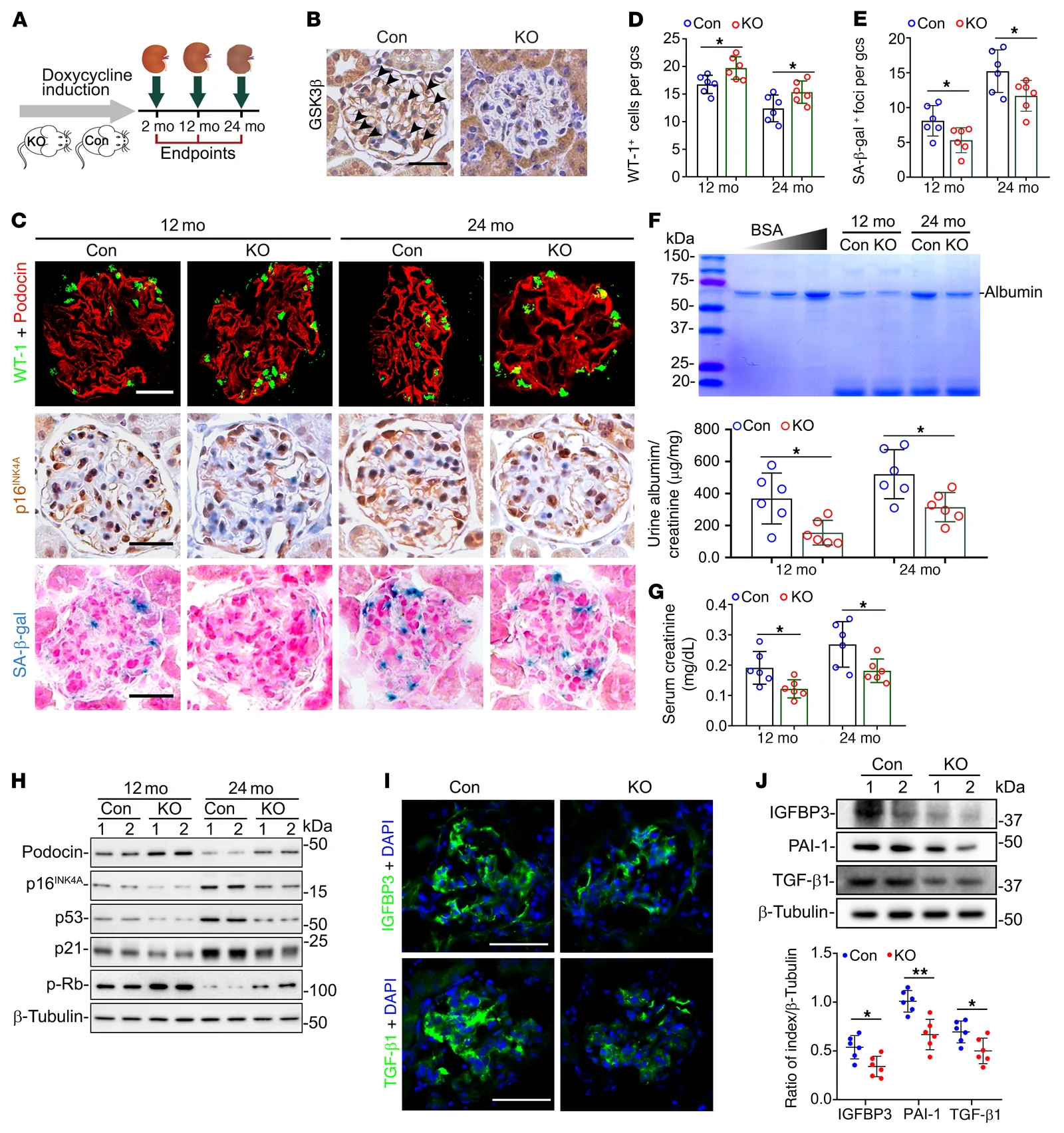
Podocyte-specific ablation of GSK3β in mice mitigates podocyte senescence and senescence-associated secretory phenotypes (SASPs) and improves kidney aging. (**A**) Schematic diagram illustrates the animal experimental design. (**B**) Kidney specimens collected from podocyte-specific GSK3β-knockout (KO) and control (Con) mice at 2 months of age (mo) were processed for peroxidase staining for GSK3β, as shown by representative micrographs. Arrowheads indicate GSK3β-positive podocytes. Scale bars: 20 μm. (**C**) Kidney specimens were subjected to fluorescent immunostaining for WT-1 and podocin, peroxidase immunostaining for p16^INK4A^, and SA-β-gal activity staining, as shown by representative micrographs. Scale bars: 20 μm. (**D** and **E**) The average number of (**D**) WT-1^+^ podocytes and (**E**) SA-β-gal^+^ foci per glomerular cross section (gcs) by absolute counting. *P < 0.05 (*n =* 6 mice per group). (**F**) Spot urine was collected at the indicated time points, and an aliquot (20 μL) was resolved by SDS-PAGE followed by Coomassie brilliant blue staining. Bovine serum albumin (BSA; 1, 2, and 4 μg) served as standard control. Urine samples were processed for albumin ELISA analysis with adjustment for creatinine concentrations. *P < 0.05 (*n =* 6). (**G**) Serum creatinine levels in KO mice were significantly lower than those in Con mice. *P < 0.05 (*n =* 6). (**H**) Representative immunoblot analysis of glomeruli isolated from Con and KO mice for indicated proteins. β-Tubulin served as a loading control. (**I**) Kidney specimens collected at 24 months were processed for fluorescent immunohistochemical staining for SASP factors. Scale bars: 50 μm. (**J**) Representative immunoblot analysis of glomeruli isolated at 24 months for SASP factors. Densitometric analyses of the expression levels of SASP factors in glomeruli, presented as relative levels normalized to β-tubulin based on immunoblot analysis. *P < 0.05, **P < 0.01 (*n =* 6). Data are expressed as mean ± SD. Panels **D**–**G** and **J** were analyzed by 2-tailed, unpaired Student’s *t* test.

To determine whether GSK3β overexpression actually mediates podocyte senescence and glomerular aging, we studied transgenic mice with doxycycline-inducible podocyte-specific KO of the GSK3β gene (23). Mice with the KO genotype were fed with doxycycline to induce the ablation of GSK3β and then maintained with ad libitum access to water and standard chow. Littermates with control genotypes (Con) were similarly treated (Figure 3A). Peroxidase immunohistochemical staining of murine kidney tissues at 2 months of age revealed that GSK3β expression was selectively suppressed in the periphery of glomerular tufts in KO mice (Figure 3B), consistent with successful ablation of GSK3β in podocytes. In line with the above observations made in WT mice, signs of kidney aging, including extracellular matrix accumulation in glomeruli and the renal interstitium, increased numbers of globally sclerotic glomeruli (Supplemental Figure 4, A and B), broadening of podocyte foot processes (Supplemental Figure 4, C and D), and amplified expression of fibronectin in glomeruli (Supplemental Figure 4, C and E) were observed in Con mice as early as 12 months of age and were more pronounced at 24 months of age. In contrast, all these features of kidney aging were attenuated in KO mice at both 12 and 24 months. In addition, glomerular staining for the senescence marker p16^INK4A^ and SA‑β‑gal activity were also reduced in KO mice as compared with Con mice (Figure 3, C and E). This was associated with less podocyte loss and degeneration in KO mice, as shown by immunostaining for WT-1 and podocin (Figure 3, C and D), along with reductions in albuminuria (Figure 3F) and preservation of kidney function, as reflected by serum creatinine levels (Figure 3G). The improved kidney aging in KO mice, relative to Con mice, appeared to be causally related to reduction in the senescence signaling activity in glomeruli, because immunoblot analysis of isolated glomeruli revealed that KO mice had suppressed expression of p16^INK4A^, p53, and p21, but augmented expression of p-Rb, which was associated with more podocin expression as compared with Con mice (Figure 3H). In parallel, glomerular expression of the subset of SASP factors implicated in fibrogenesis, TGF-β1, IGFBP3, and PAI-1, was diminished in KO mice (Figure 3, I and J).

Some senescence signaling mediators may also regulate the cell cycle (5). To determine whether cell cycle events in the kidney were altered in KO mice, kidney sections were stained for a variety of cell cycle markers, including Ki67, a marker for cell cycle entry, as well as proliferating cell nuclear antigen (PCNA) and phospho-histone H3 (p-H3), putative markers respectively for S and G_2_/M phases. As shown in Supplemental Figure 5, A–D, very few cells in glomeruli and a small number of renal tubular cells were positive for Ki67 that was similarly expressed in Con and KO mice. These proliferative cells were either in the S phase as probed by PCNA or in the G_1_ phase, which was quantified by subtracting the number of cells in the S or G_2_/M phase from the total number of proliferating cells positive for Ki67. Cells in the G_2_/M phase that were positive for p-H3 were rare in renal tubulointerstitium or glomeruli (Supplemental Figure 5, A, E, and F). In contrast, proliferating podocytes, marked by positivity for both WT-1 and Ki67 or other cell cycle markers in glomeruli, were barely detected in both Con and KO mice (Supplemental Figure 5, A and D). These findings suggest that there is very minimal proliferation of glomerular podocytes during the aging process, as would be expected in terminally differentiated postmitotic cells (33).

### GSK3β is essential and sufficient for senescence signaling in glomerular podocytes.

**Figure 4:**
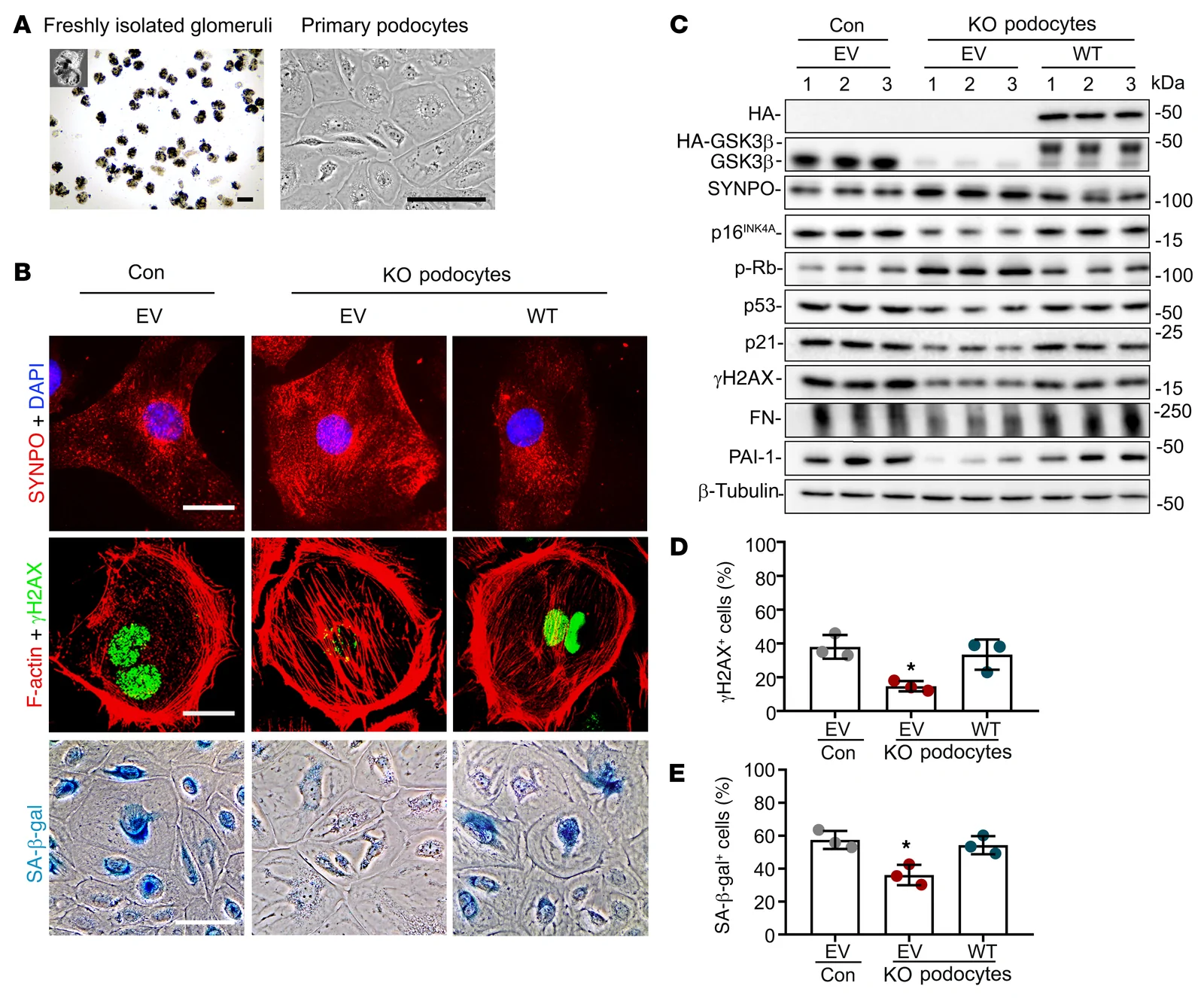
Cellular senescence and senescence-associated secretory phenotypes (SASPs) are mitigated in primary podocytes derived from KO mice and reinstated after GSK3β reconstitution. (**A**) Primary podocytes were cultured from glomeruli isolated from 12-month-old control mice (Con) and mice with podocyte-specific GSK3β knockout (KO). Representative micrographs show freshly isolated glomeruli and primary cultures of podocytes. Scale bars: 75 μm. (**B**–**E**) Primary podocytes were subjected to electroporation-based transfection with either an empty plasmid vector (EV) or a plasmid encoding the HA-conjugated WT GSK3β by using the Amaxa Nucleofection kit. (**B**) Cells were processed for SA-β-gal activity staining or immunofluorescent staining for synaptopodin (SYNPO; red) or γH2AX (green) followed by counterstaining with DAPI for nuclei or with rhodamine-phalloidin for F-actin (red). Scale bars: 20 μm (top 2 rows) and 30 μm (bottom row). (**C**) Cell lysates were processed for immunoblot analysis for indicated proteins, including SASP factors like fibronectin (FN) and PAI-1. β-Tubulin served as a loading control. (**D**) Absolute count of the number of γH2AX^+^ cells expressed as percentages of the total number of cells per microscopic field. *P < 0.05 versus all other groups (*n =* 3). (**E**) Quantification of the SA-β-gal^+^ cells as percentages of the total number of cells per microscopic field. *P < 0.05 versus all other groups (*n =* 3). Data are expressed as mean ± SD. Panels **D** and **E** were analyzed by 1-way ANOVA followed by Tukey’s test.

To examine the role of GSK3β in senescence signaling in podocytes, primary cultures of podocytes were prepared from glomeruli isolated from KO and Con mice aged 12 months (Figure 4A). Primary podocytes were then subjected to electroporation-based transfection with an empty plasmid vector (EV) or plasmids encoding a hemagglutinin-tagged (HA-tagged) WT GSK3β or dominant-negative kinase-dead (KD) mutant GSK3β, with a satisfactory transfection efficiency, as confirmed by fluorescent immunocytochemical staining for HA (Supplemental Figure 6A). Consistent with the in vivo findings, senescence was significantly reduced in primary KO podocytes as compared with Con podocytes, as evidenced by staining for SA-β-gal activity or γH2AX (Figure 4B), a molecular marker for DNA double-strand breaks and aging (31), followed by absolute counting and quantification (Figure 4, D and E). Primary KO podocytes also displayed less podocyte degenerative changes, marked by reduced expression of the podocyte homeostatic marker synaptopodin, and disruption of the actin cytoskeleton as assessed by phalloidin staining (Figure 4B). The beneficial effects of GSK3β KO seemed to be mediated by suppressed senescence signaling, as shown by reduced expression of p16^INK4A^, p53, and p21, and increased expression of p-Rb, as well as diminished expression of fibrogenic SASP factors PAI-1 and fibronectin, as compared with Con podocytes similarly subjected to EV transfection (Figure 4C and Supplemental Figure 6B). In contrast, reconstitution of WT GSK3β (Figure 4C and Supplemental Figure 6B) but not KD GSK3β (Supplemental Figure 6C) in KO podocytes restored the senescence signaling activity and degenerative changes (Figure 4B). Despite the differences in senescence signaling, no significant differences in cell cycle profiles were observed between primary KO podocytes and Con podocytes, as shown by flow cytometric analysis (Supplemental Figure 7, A and B).

### p16^INK4A^ and p53, key mediators of senescence signaling, colocalize and physically interact with GSK3β as its putative substrates in glomerular podocytes.

**Figure 5:**
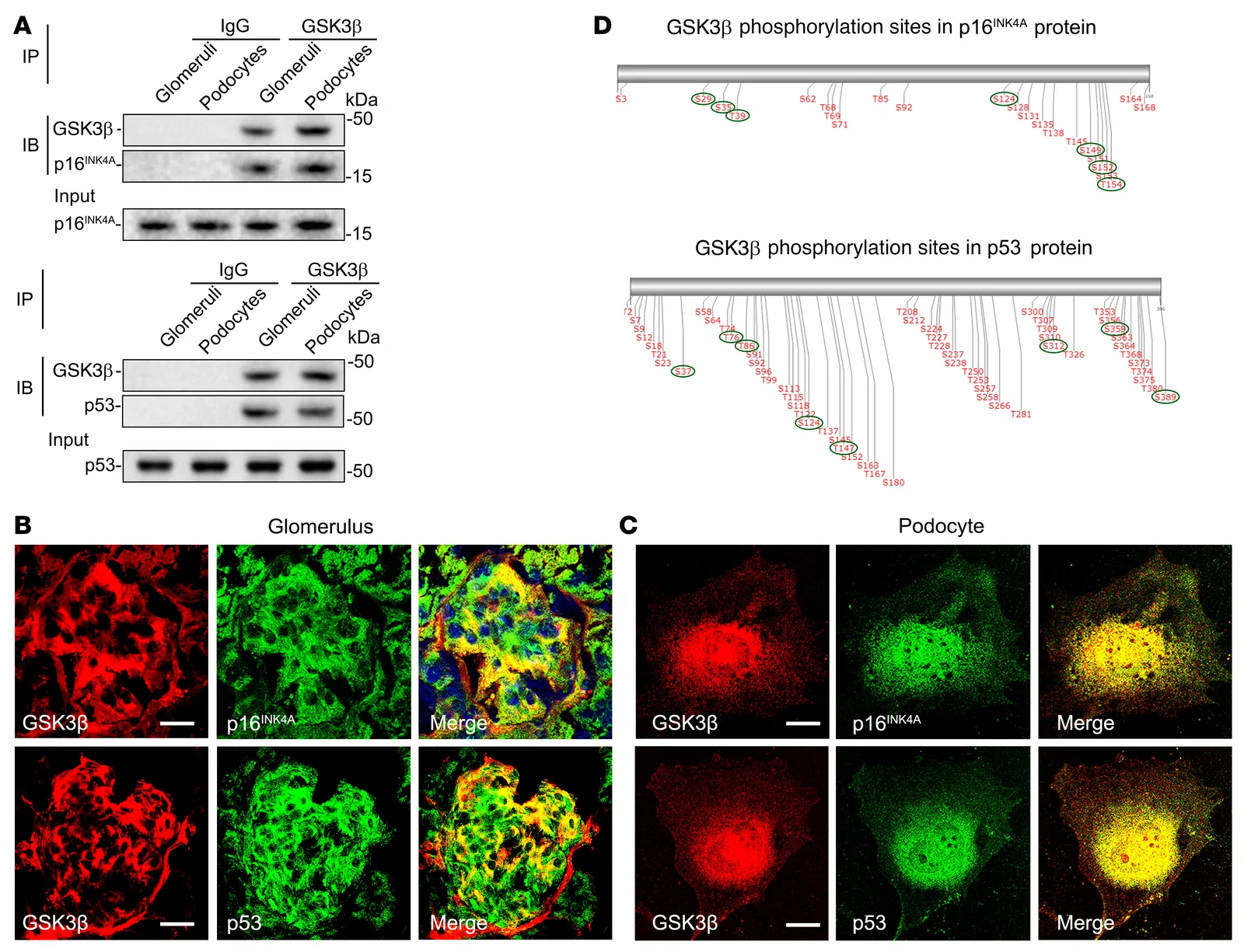
p16^INK4A^ and p53 colocalize and physically interact with GSK3β in glomerular podocytes as its putative substrates. (**A**) Lysates of differentiated immortalized murine podocytes and homogenates of glomeruli isolated from WT mice were processed for immunoprecipitation (IP) by using an anti-GSK3β antibody or preimmune IgG, followed by immunoblot analysis (IB) of immunoprecipitates for GSK3β, p16^INK4A^, and p53 in parallel with input controls. (**B** and **C**) Dual-color fluorescent immunostaining for GSK3β (red) and p16^INK4A^ (green) or p53 (green) in (**B**) mouse kidney tissues as revealed by fluorescence microscopy or in (**C**) cultured murine podocytes as shown by laser scanning confocal fluorescence microscopy. Scale bars: 20 μm. (**D**) In silico analysis reveals serine/threonine residues in putative consensus motifs for phosphorylation by GSK3β in p16^INK4A^ and p53. The serines and threonines with high prediction scores for GSK3β consensus motifs are marked with green circles.

To further examine the molecular mechanisms underlying the GSK3β modulation of senescence signaling, the physical interaction between GSK3β and diverse senescence signaling molecules were examined in isolated glomeruli and cultured podocytes. Homogenates of isolated glomeruli and lysates of cultured murine podocytes were processed for immunoprecipitation by using an anti-GSK3β antibody followed by immunoblot analysis for a number of selected senescence signaling transducers, including p16^INK4A^, p53, p21, p19, Rb, CDK2, and CDK4 (34, 35). As shown in Figure 5A, among these senescence mediators, p16^INK4A^ and p53 evidently coprecipitated with GSK3β in both glomerular homogenates and podocyte lysates. To further validate this finding, mouse kidney sections and cultured podocytes were examined by dual-color immunofluorescent staining for GSK3β and p16^INK4A^ or p53 (Figure 5, B and C). Once again, p16^INK4A^ or p53 colocalized with GSK3β staining in the periphery of glomerular tufts, consistent with podocyte localization. Moreover, in cultured podocytes, a discrete area of GSK3β staining distributed mainly in the cytoplasm and, to a lesser extent, in nuclei, colocalized with p16^INK4A^ or p53, as shown by laser scanning confocal fluorescence microscopy. These findings suggest that GSK3β may physically interact with p16^INK4A^ and p53. A growing body of evidence suggests that p16^INK4A^ and p53 undergo posttranslational regulation, including phosphorylation (36–39). Indeed, phosphorylation of p16^INK4A^ and p53 at serine 152 and 37, respectively, is required for the prosenescence signaling activities (38, 40, 41). As a ubiquitously expressed serine/threonine kinase, GSK3β is known to catalyze the phosphorylation of a number of substrate proteins (20). To further test whether GSK3β regulates p16^INK4A^ and p53 phosphorylation, the amino acid sequences of p16^INK4A^ and p53 were examined by computational active site analysis for putative consensus phosphorylation motifs for GSK3β. As shown in Figure 5D, in silico analysis revealed that a number of amino acid residues in p16^INK4A^ and p53 reside in the consensus motifs for phosphorylation by GSK3β with statistically significant prediction scores, suggesting that p16^INK4A^ and p53 are putative substrates for GSK3β.

**Figure 6:**
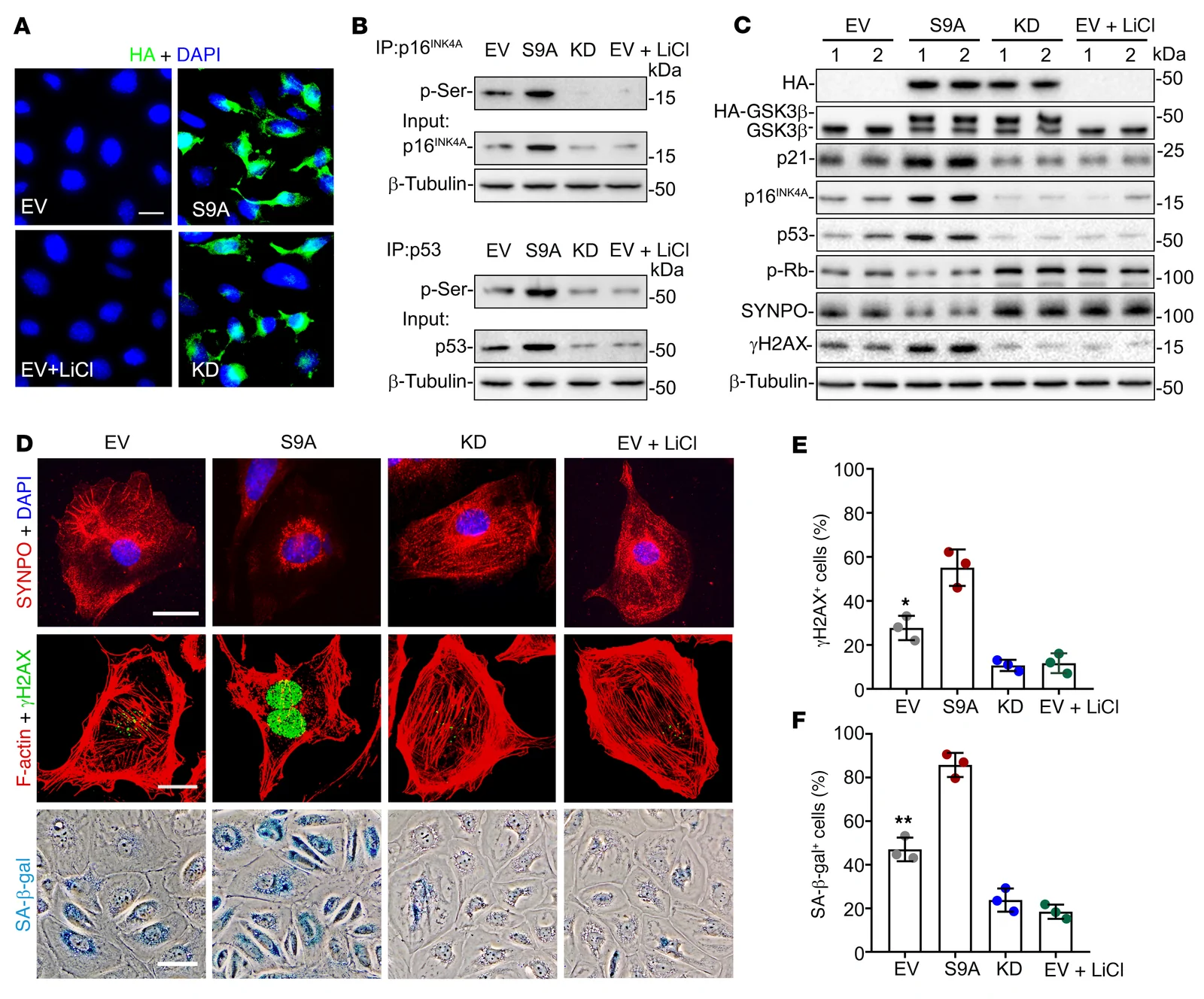
GSK3β regulates the phosphorylation of p16^INK4A^ and p53, resulting in modulation of senescence signaling in podocytes. Conditionally immortalized murine podocytes were transiently lipotransfected with a control empty plasmid vector (EV), or plasmids encoding the HA-conjugated dominant-negative kinase dead (KD) mutant of GSK3β or constitutively active (S9A) mutant of GSK3β in the presence or absence of lithium chloride (LiCl, 10 mM) or an equal volume of vehicle. (**A**) After different treatments, cells were subjected to immunofluorescent staining for HA, which revealed a transfection efficiency of approximately 80%. Scale bar: 20 μm. (**B**) Whole cell lysates were processed for immunoprecipitation (IP) by using an anti-p16^INK4A^ or -p53 antibody, followed by immunoblot analysis (IB) of immunoprecipitates for phosphorylated serine (p-Ser), in parallel with input controls. (**C**) Representative immunoblot analysis of cell lysates for indicated molecules. β-Tubulin served as a loading control. (**D**) Cells were subjected to SA-β-gal activity staining, or to immunofluorescent staining for synaptopodin (SYNPO; red) or γH2AX (green) followed by counterstaining with DAPI for nuclei or with rhodamine-phalloidin for F-actin (red). Scale bars: 20 μm. (**E**) Absolute count of the number of γH2AX^+^ cells as percentages of the total number of cells per microscopic field. *P < 0.05 versus all other groups (*n =* 3). (**F**) Quantification of the SA-β-gal^+^ cells as percentages of the total number of cells per microscopic field. **P < 0.01 versus all other groups (*n =* 3). Data are expressed as mean ± SD. Panels **E** and **F** were analyzed by 1-way ANOVA followed by Tukey’s test.

To further validate the role of GSK3β in the regulation of cellular senescence signaling, GSK3β activity was specifically manipulated in murine podocytes by ectopic expression of a constitutively active (S9A) or the KD GSK3β mutant, or by lithium, a chemical inhibitor of GSK3β. Fluorescent immunocytochemical staining confirmed satisfactory and comparable lipofectamine-based transfection of podocytes with plasmids encoding S9A or KD (Figure 6A). The phosphorylation of serine residues in p16^INK4A^ and p53 was increased in podocytes expressing S9A but abrogated in KD-expressing or lithium-treated cells (Figure 6B). Importantly, senescence signaling was induced in S9A-expressing podocytes, marked by increased expression of p16^INK4A^, p53, and p21, and reduced expression of p-Rb (Figure 6C and Supplemental Figure 8), resulting in amplified podocyte senescence, as probed by γH2AX or SA-β-gal activity staining (Figure 6, D–F). Enhanced podocyte senescence was associated with podocyte degenerative changes, characterized by diminished expression of synaptopodin and disruption of the phalloidin-labeled actin cytoskeleton (Figure 6D). Conversely, KD overexpression suppressed senescence signaling and podocyte senescence and degeneration (Figure 6, C–F).

### Therapeutic targeting of GSK3β by microdose lithium intercepts the senescence signaling pathway and attenuates kidney aging in mice.

**Figure 7:**
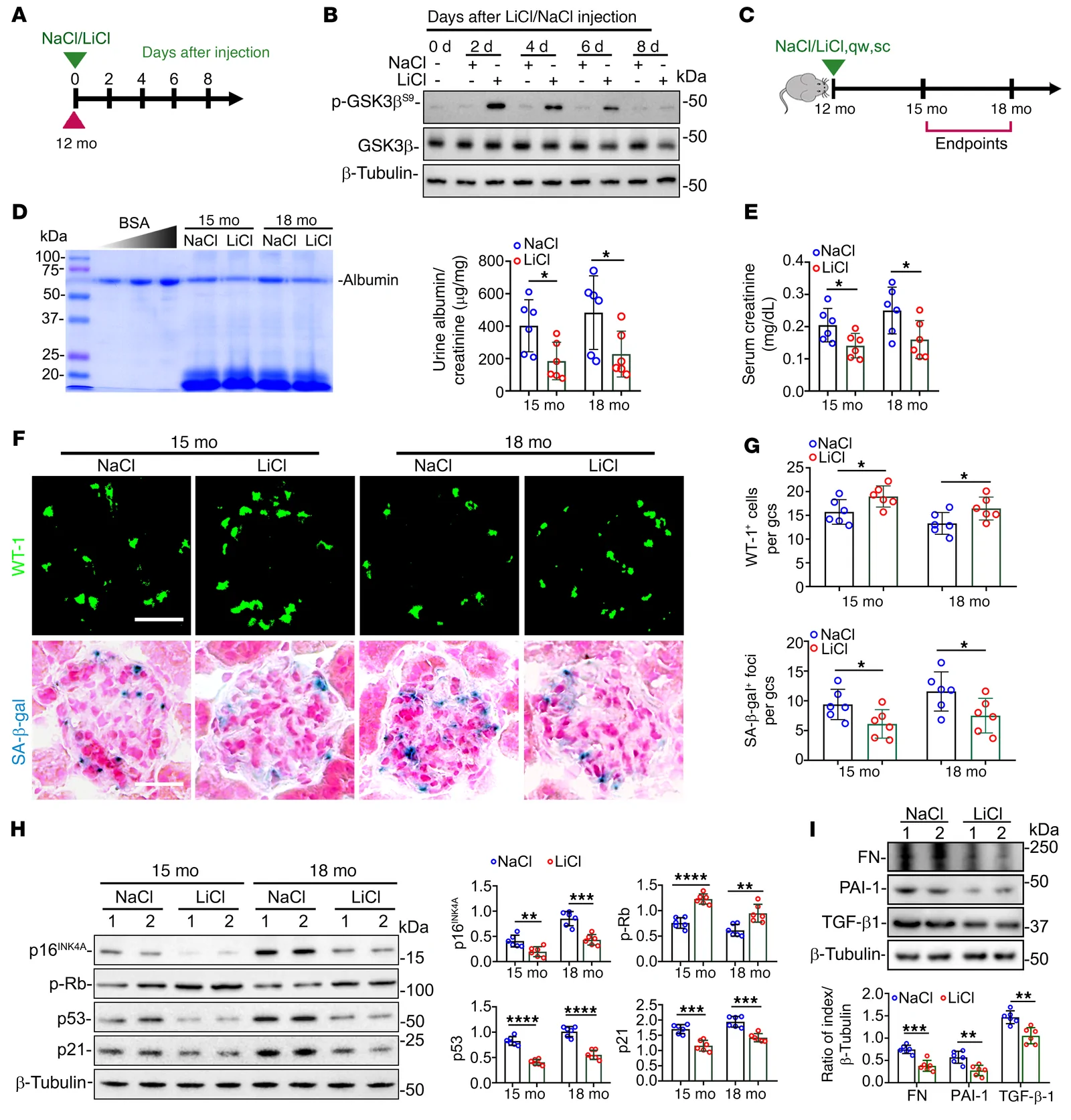
Once-weekly microdose lithium treatment later in life suppresses podocyte senescence and senescence-associated secretory phenotypes (SASPs) in mice, resulting in a retarded renal aging. (**A**) Schematic diagram illustrates the pilot experiment to optimize the regimen of lithium therapy in mice. (**B**) On indicated days (d) after LiCl or NaCl treatment, protein was extracted from whole kidneys (pool of 3 animals per group) for immunoblot analysis for indicated molecules. (**C**) Schematic diagram illustrates the experimental design in WT aging mice. (**D**) Spot urine was collected at the indicated month (mo) and an aliquot (20 μL) was resolved by SDS-PAGE followed by Coomassie brilliant blue staining. Bovine serum albumin (BSA; 1, 2, and 4 μg) served as standard control. Urine samples were processed for albumin ELISA analysis with adjustment for creatinine concentrations. *P < 0.05 (*n =* 6). (**E**) Renal function was assessed by serum creatinine levels. *P < 0.05 (*n =* 6). (**F**) Kidney specimens were subjected to immunofluorescent staining for WT-1 or SA-β-gal activity staining. Scale bars: 20 μm. (**G**) The average number of WT-1^+^ cells and SA-β-gal^+^ foci per glomerular cross section (gcs) by absolute counting. *P < 0.05 (*n =* 6 mice per group). (**H**) Representative immunoblot analysis of isolated glomeruli. Densitometric analyses of the expression levels of indicated proteins, presented as relative levels normalized to β-tubulin based on immunoblot analysis. **P < 0.01, ***P < 0.001, ****P < 0.0001 (*n =* 6). (**I**) Representative immunoblot analysis of glomeruli isolated at 18 months for SASP factors fibronectin (FN), PAI-1, and TGF-β1. Densitometric analyses of the expression levels of indicated proteins, presented as relative levels normalized to β-tubulin based on immunoblot analysis. **P < 0.01, ***P < 0.001 (*n =* 6). Data are expressed as mean ± SD. Panels **D**, **E**, and **G**–**I** were analyzed by 2-tailed, unpaired Student’s *t* test.

To examine whether GSK3β-regulated senescence signaling could provide a therapeutic target for modifying the kidney aging process, aging mice were treated with lithium, a known inhibitor of GSK3β. Of note, the dose of lithium needed to attain effects on peripheral organs is much less than the psychiatric dose (18). A pilot study was performed to determine the lowest optimal dose of lithium that could effectively block the activity of GSK3β in the aging kidney (Figure 7A). WT mice aged 12 months received a single microdose of lithium chloride (40 mg/kg, s.c.) or equal molar amounts of sodium chloride as controls. Mice were then sacrificed every other day. Immunoblot analysis of whole kidney homogenates demonstrated that lithium markedly induced inhibitory phosphorylation of GSK3β^S9^, whereas sodium chloride had no effect. This effect of lithium gradually waned, and by day 8, renal expression of phosphorylated GSK3β^S9^ was almost comparable to that on day 0 (Figure 7B). Accordingly, once-weekly injection of microdose lithium or sodium was used in subsequent studies and administered to mice aged 12 months (Figure 7C). After 3 or 6 months of treatment, lithium but not sodium treatment markedly ameliorated albuminuria, as shown by urine protein electrophoresis and by urinary albumin-to-creatinine ratios (Figure 7D), and improved kidney function, as assessed by serum creatinine levels (Figure 7E). In addition, lithium treatment significantly attenuated podocytopenia, characterized by diminished WT-1–positive cells per glomerular cross section, and mitigated cellular senescence in glomeruli, marked by SA-β-gal activity staining (Figure 7, F and G). Mechanistically, senescence signaling activity in isolated glomeruli was abrogated by lithium treatment, as evidenced by repressed expression of p16^INK4A^, p53, and p21, along with elevated expression of p-Rb, as well as diminished production of SASP factors TGF-β1, PAI-1, and fibronectin, as shown by immunoblot analysis in combination with densitometry (Figure 7, H and I). Moreover, as shown by staining for Ki67 (Supplemental Figure 9, A–D), lithium treatment slightly increased the number of proliferative cells in renal tubules, which were mostly in G_1_ and S phases (Supplemental Figure 9, C and F). Nevertheless, the number of glomerular cells or podocytes positive for Ki67 or any other cell cycle markers was similar in kidneys from lithium- or sodium-treated mice (Supplemental Figure 9, B, D, and E), suggesting that lithium had minimal effects on podocyte proliferation or cell cycle profiles during aging.

### GSK3β-regulated cellular senescence in renal tubules.

In addition to glomeruli, increased expression of GSK3β was also detected focally in cortical tubules in aging kidneys in humans (Figure 1C). In mice, the expression and activity of GSK3β likewise increased focally in cortical renal tubules with age, as determined by immunohistochemical staining and by immunoblot analysis of whole kidney specimens (Supplemental Figure 10, A and B). This was associated with focally increased renal tubular expression of senescence mediators p16^INK4A^ and p53, suggesting that GSK3β-regulated senescence signaling is also operative and contributes to aging in other compartments of the kidney. Renal tubular overexpression of GSK3β and senescence markers was marginally reduced by podocyte-specific ablation of GSK3β in aging mice (Supplemental Figure 10, C and D), possibly secondary to prevention of glomerular aging and proteinuria. However, they were substantially mitigated by microdose lithium treatment (Supplemental Figure 10, E and F), suggesting that GSK3β-promoted cellular senescence could also be a pharmacological target in other compartments of the kidney.

### Lithium therapy in psychiatric patients inhibits GSK3β and mitigates senescence signaling in podocytes, concomitant with better-preserved kidney function.

**Figure 8:**
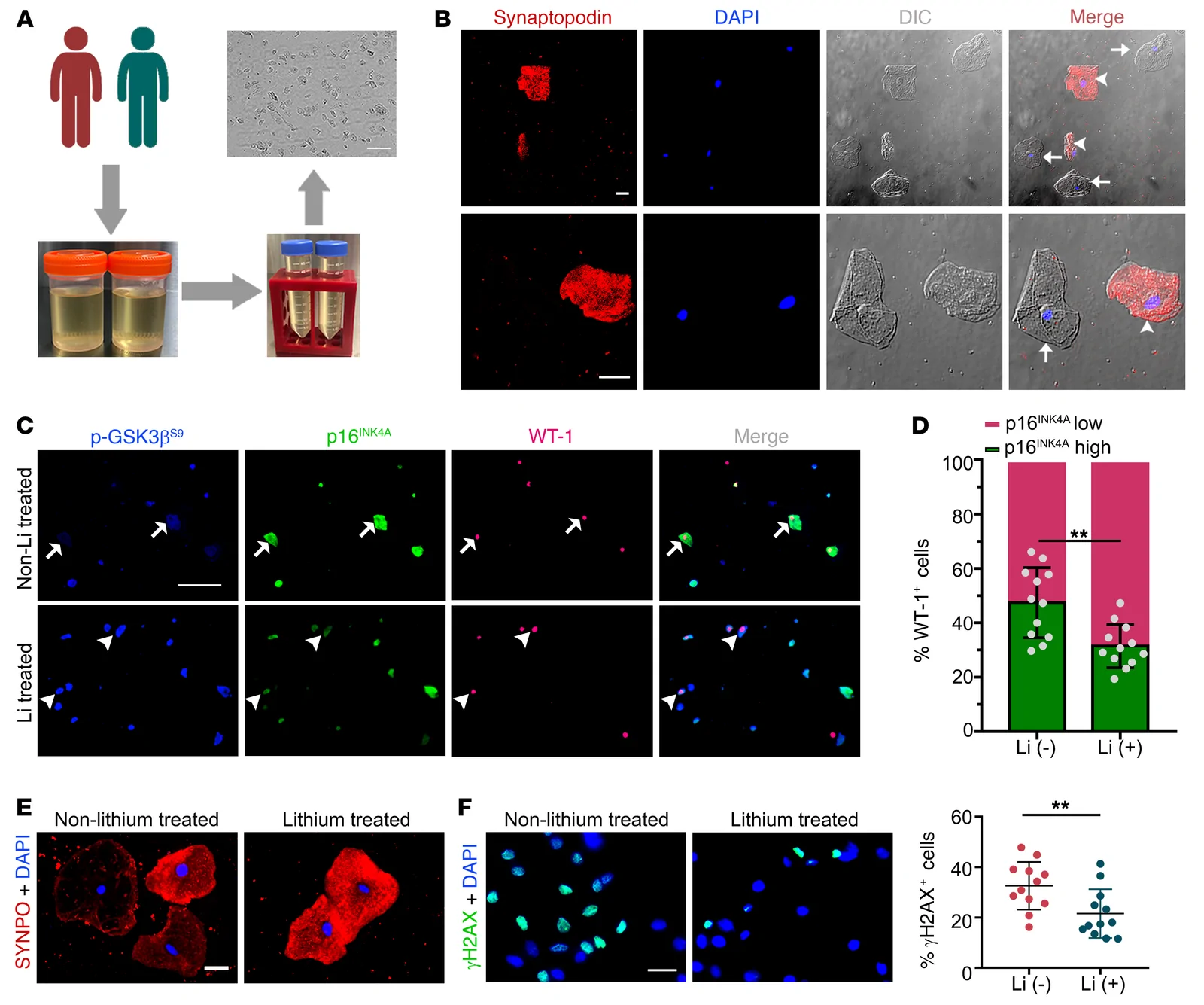
Long-term lithium carbonate therapy in psychiatric patients inhibits GSK3β activity and attenuates cellular senescence in urinary exfoliated cells. (**A**) Schematic diagram depicts preparation of urinary exfoliated cells from psychiatric patients treated either with lithium carbonate [Li (+), *n =* 12] or without lithium carbonate [Li (–), *n =* 12]. Scale bars: 100 μm. (**B**) Immunofluorescent staining of urinary exfoliated cells for synaptopodin (red) with DAPI counterstaining for nuclei, as shown by fluorescence microscopy and differential interference contrast (DIC) microscopy. Arrowheads indicate synaptopodin-positive podocytes, while arrows indicate synaptopodin-negative urinary cells. Scale bars: 20 μm. (**C**) Multicolor immunofluorescent staining of urinary exfoliated cells for phosphorylated GSK3β at serine 9 (p-GSK3β^S9^), p16^INK4A^, and WT-1. Arrows indicate WT-1–positive urinary podocytes with p-GSK3β^S9-lo^p16^hi^ staining pattern. Arrowheads indicate WT-1–positive urinary podocytes with p-GSK3β^S9-hi^p16^lo^ staining pattern. Scale bars: 100 μm. (**D**) Quantification of cells with high and low expression of p16^INK4A^ among all WT-1^+^ urinary cells. **P < 0.01 (*n =* 12). (**E**) Immunofluorescent staining of urinary exfoliated cells for synaptopodin (SYNPO) followed by counterstaining with DAPI. Scale bars: 20 μm. (**F**) Immunofluorescent staining of urine exfoliated cells for γH2AX followed by counterstaining with DAPI. Scale bars: 20 μm. Absolute count of the number of γH2AX-positive cells as percentage of the number of urinary exfoliated cells per microscopic field. **P < 0.01 (*n =* 12). Data are expressed as mean ± SD. Panels **D** and **F** were analyzed by 2-tailed, unpaired Student’s *t* test.

To determine whether lithium alters GSK3β-regulated senescence signaling and kidney aging in humans, a group of psychiatric patients who received long-term treatment with lithium carbonate was studied. All patients had received routine therapeutic drug monitoring of lithium and had no evidence of lithium overdose or toxicity. Another group of psychiatric patients, who were matched for age, sex, and duration of mental diseases but never treated with lithium carbonate, served as controls (Supplemental Table 1). Turnover and shedding of renal epithelial cells, such as glomerular podocytes, is a normal ongoing process and is associated with continuous excretion of renal parenchymal cells into the urine (42). Exfoliated cells in the urine have been successfully utilized as a type of noninvasive liquid kidney biopsy for research and diagnostic purposes (42, 43), and were collected in our patients (Figure 8, A and B). As shown in Figure 8C, multicolor fluorescent immunocytochemical staining demonstrated that the level of inhibitory phosphorylation of GSK3β^S9^ was inversely associated with that of the prosenescence signaling mediator p16^INK4A^ in urinary exfoliated cells, in support of the hypothesis that GSK3β also modulates senescence signaling in humans. This morphologic finding was corroborated by statistical analysis of individual urinary exfoliated cells categorized based on 4 patterns of staining, i.e., p16^hi^p-GSK3β^S9-lo^, p16^hi^p-GSK3β^S9-hi^, p16^lo^p-GSK3β^S9-lo^, and p16^lo^p-GSK3β^S9-hi^ (Table 1). Lithium-treated patients tended to excrete more of the p16^lo^p-GSK3β^S9-hi^ cells expressing high levels of phosphorylated GSK3β^S9^ and low levels of p16^INK4A^, consistent with an antiaging activity (Table 1). Moreover, in urinary WT-1–positive podocytes derived from lithium-treated patients, the level of phosphorylated GSK3β^S9^ was also enhanced as expected (Figure 8C), and associated with reduced expression of p16^INK4A^ (Figure 8D). In parallel, podocyte degeneration was attenuated in lithium-treated patients, marked by retention of the podocyte homeostatic marker synaptopodin (Figure 8E), whereas urinary exfoliated cells positive for the senescence marker γH2AX (Figure 8F) were diminished, suggesting that lithium therapy confers a rejuvenating effect on kidney cells, including podocytes. Indeed, lithium-treated patients demonstrated a trend toward better kidney functions as indicated by preservation of eGFR and lower urinary albumin-to-creatinine ratios (Supplemental Table 1), despite comparable age, sex, duration of mental disease, and comorbidities.

## Discussion

Kidney aging is an emerging challenge. It impairs kidney function and predisposes older people to AKI, CKD, and other diseases (1, 3). As yet, there are no treatments to slow or halt kidney aging. This study demonstrates that GSK3β is a key regulator of senescence signaling in the kidney. Genetic or pharmacologic targeting of GSK3β in podocytes attenuates senescence and SASP in glomeruli and mitigates kidney aging. Our findings have significant therapeutic potential because lithium, a standard inhibitor of GSK3β, is an FDA-approved agent for treating affective mental illness (18). If validated in large clinical trials, low-dose lithium might also be an effective antiaging medication for the kidney and, potentially, other organ systems.

A beneficial effect of low-dose lithium on aging has also been reported in experimental organisms like *C*. *elegans* and flies (7–9), and is supported by epidemiologic evidence in humans (7, 11). In rodent models, microdose lithium conferred protection against AKI (24, 44–46), CKD (27, 47), and glomerular injury (48, 49). However, clinical use of lithium in psychiatric patients has been reported to have adverse renal effects and deleterious clinical consequences, including nephrogenic diabetes insipidus, which is attributed to effects of high-dose lithium on distal renal tubules and often resolves after cessation of lithium (50). Nevertheless, the bulk of epidemiologic data suggest lithium nephrotoxicity is actually uncommon and usually associated with high serum levels of lithium and longer duration of lithium therapy (18, 51). Of note, comparatively high doses of the lipophobic lithium ion are required to reach therapeutic levels in cerebrospinal fluid and treat mental diseases (18, 52). Accordingly, the psychiatric dose of lithium is much greater than that associated with beneficial effects on peripheral organs like the kidney. While the optimal dose of lithium for kidney protection is still unknown, evidence suggests that a dose less than one-third of the neurobiological dose seems to be sufficient to block renal GSK3β activity in animal models (18). As shown in this and other studies, this low dose has significant effects on diverse signaling pathways (18, 24, 49), and on kidney aging, with no noticeable adverse effects.

Besides the senescence signaling pathway, GSK3β has many other targets, with β-catenin being a major one (20). Although β-catenin is essential for podocyte homeostasis (53), excessive β-catenin activation may contribute to podocyte injury (54). Conditional ablation of GSK3β in podocytes, however, barely affected the β-catenin pathway and was not associated with any renal phenotypes in young mice, as shown by us (21) and by Hurcombe et al. (22). This is probably attributable to compensation for the loss of GSK3β by GSK3α, which seems to be redundant with GSK3β in mediating Wnt/β-catenin signaling (55). In support of this, Hurcombe et al. (22) demonstrated that podocyte-specific KO of both GSK3α and GSK3β in embryonic or adult mice activated β-catenin and disrupted Hippo signaling in podocytes, resulting in severe podocyte injury, glomerulosclerosis, and heavy proteinuria. Consistently, by using compound KOs of GSK3α and -β, Doble et al. (55) also demonstrated that genetic deletion of at least 3 of the 4 alleles of both isoforms of GSK3 is required to cause an appreciable change in β-catenin activity, suggesting that there may be a therapeutic window within which chemical inhibitors can effectively block GSK3β signaling without altering β-catenin levels in peripheral organs, like the kidney. Our data suggest that microdose lithium may be one such inhibitor to satisfy this need.

Global glomerulosclerosis is the histological hallmark of kidney aging (1, 2). Podocyte inadequacy due to senescence and loss together with glomerular fibrogenesis induced by SASP paracrine signaling is a fundamental pathogenic mechanism that promotes age-related glomerulosclerosis (56, 57). Glomerular podocytes are highly specialized and terminally differentiated cells with limited capacity to proliferate (33). In fact, the beneficial effects of GSK3β KO or inhibition in glomerular aging were not associated with significant podocyte proliferation. In addition to podocyte senescence, age-related changes in other compartments of the kidney may also contribute to kidney aging. In particular, renal tubular atrophy is another hallmark of kidney aging and was observed in the present study and associated with GSK3β overexpression and senescence in renal tubular epithelial cells. In light of the finding that renal progenitors with the potential to regenerate glomerular podocytes (58) are physically located in renal proximal tubules (59), it is conceivable that targeting GSK3β-regulated senescence in renal tubules may spare renal progenitors, promote podocyte regeneration, and thereby attenuate glomerular and kidney aging. Indeed, the beneficial effect of lithium therapy on renal aging coincided with reinforced cell cycle progression in cortical renal tubular cells.

How does GSK3β modulate podocyte senescence? Our data suggested that GSK3β may have a direct effect on senescence signaling by interacting with key senescence signaling mediators like p16^INK4A^ and p53, which are putative substrates for GSK3β. Thus, it is conceivable that age-related GSK3β hyperactivity increases phosphorylation of p16^INK4A^ and p53, which is required for their prosenescence activities (37–39, 58), thereby promoting senescence signaling activity and kidney aging. It is possible that GSK3β may target other substrates and signaling pathways in addition to p16^INK4A^ and p53 (20). Consistent with this speculation, Nrf2, mTOR, AMPK, and other mediators have also been implicated in modifying the senescence signaling pathway (4, 5). Our finding by no means rules out the contribution of other GSK3β-regulated pathways to kidney aging.

In summary, the senescence signaling pathway in glomerular podocytes is regulated by GSK3β, which shows increased expression and activity with age. Targeted inhibition of GSK3β in podocytes via conditional KO or pharmacologic blockade with microdose lithium is able to intercept the senescence signaling, mitigate podocyte senescence, and slow kidney aging. Our findings suggest that GSK3β-regulated senescence signaling may be an attractive target for therapeutic interventions aimed at delaying kidney aging.

## Methods

### Antibodies and plasmids.

The following antibodies were purchased from commercial vendors: mouse anti-GSK3β (sc-377213, Santa Cruz Biotechnology); rabbit anti-GSK3β (9315, Cell Signaling Technology); rabbit anti-GSK3β (ab32391, Abcam); rabbit anti-p16^INK4A^ (ab189034, Abcam); mouse anti-p16^INK4A^ (sc-1661, Santa Cruz Biotechnology); mouse anti–WT-1 (sc-7385, Santa Cruz Biotechnology); goat anti–WT-1 (sc-15421, Santa Cruz Biotechnology); mouse anti–phosphorylated GSK3β at serine 9 (anti–p-GSK3β^S9^) (sc-373800, Santa Cruz Biotechnology); rabbit anti–p-GSK3β^S9^ (9336, Cell Signaling Technology); mouse anti–p-Rb (sc-377527, Santa Cruz Biotechnology); rabbit anti-p53 (sc-6243, Santa Cruz Biotechnology); mouse anti-p53 (sc-126, Santa Cruz Biotechnology); mouse anti-p21 (sc-6246, Santa Cruz Biotechnology); mouse anti-GAPDH (sc-32233, Santa Cruz Biotechnology); mouse anti-IGFBP3 (sc-365936, Santa Cruz Biotechnology); rabbit anti–PAI-1 (sc-8979, Santa Cruz Biotechnology); rabbit anti–TGF-β1 (sc-146, Santa Cruz Biotechnology); rabbit anti–β-tubulin (2128, Cell Signaling Technology); goat anti-podocin (sc-22298, Santa Cruz Biotechnology); rabbit anti-HA (3724, Cell Signaling Technology); rabbit anti-γH2AX (9718, Cell Signaling Technology); goat anti-synaptopodin (sc-21537, Santa Cruz Biotechnology); mouse anti-synaptopodin (sc-515842, Santa Cruz Biotechnology); rabbit anti-fibronectin (sc-9068, Santa Cruz Biotechnology); rabbit anti-fibronectin (ab2413, Abcam); rabbit anti-p19 (ab80, Abcam); rabbit anti-CDK2 (ab32147, Abcam); rabbit anti-CDK4 (ab199728, Abcam); rabbit anti–phosphorylated serine (ab9332, Abcam); rabbit anti-Ki67 (ab16667, Abcam); rabbit anti-PCNA (13110, Cell Signaling Technology); rabbit anti–p-H3 (53348, Cell Signaling Technology); mouse IgG isotype control (31903, Invitrogen); rabbit IgG isotype control (02-6102, Invitrogen); goat anti–mouse IgG horseradish peroxidase–conjugated (HRP-conjugated) antibody (31432, Invitrogen); goat anti–rabbit IgG HRP-conjugated antibody (65-6120, Invitrogen); rabbit anti–goat IgG HRP-conjugated antibody (A16142, Invitrogen); donkey anti–rabbit IgG Alexa Fluor 488 (A21206, Invitrogen); chicken anti–mouse IgG Alexa Fluor 594 (A21201, Invitrogen); chicken anti–goat IgG Alexa Fluor 594 (A21468, Invitrogen); goat anti–mouse IgG Alexa Fluor 405 (A31553, Invitrogen); donkey anti–goat IgG Alexa Fluor 488 (A11055, Invitrogen); and chicken anti–mouse IgG Alexa Fluor 488 (A21200, Invitrogen).

The control empty plasmid vector and plasmids encoding WT GSK3β (HA-GSK3β/pcDNA3), the constitutively active GSK3β mutant (HA-GSK3β S9A/pcDNA3), and the KD dominant-negative GSK3β mutant (HA-GSK3β K85A/pcDNA3) were provided by Gail V.W. Johnson (University of Alabama at Birmingham, Birmingham, Alabama, USA) and Jim Woodgett (University of Toronto) as used as described previously (24).

### Animal experimental design.

The triple-transgenic mice (*NPHS2^rtTA^* *TRE^Cre^* *GSK3β^fl/fl^*) for doxycycline-inducible podocyte-specific GSK3β KO were bred as previously described (21) on a mixed genetic background of C57BL/6 × FvB. Male KO mice received doxycycline hydrochloride (TCI) treatment at 4 to 6 weeks old via drinking water (2 mg/mL with 5% sucrose, protected from light) for 2 weeks to induce podocyte-specific GSK3β ablation. Male littermates lacking any transgenes were designated as control mice (Con) and similarly treated. Transgenic mice and additional WT male mice were fed standard chow ad libitum with free access to water and were euthanized at 2, 12, or 24 months of age. In a separate experiment, WT C57BL/6 male mice aged 12 months were randomized to receive once-a-week subcutaneous injections of lithium chloride (40 mg/kg) or an equal molar amount of sodium chloride as controls. Mice were euthanized on 0, 2, 4, 6, 8 days, or 3 or 6 months after the first injection. Spot urine, blood, and kidney tissues were collected for further examinations. Six mice were randomly assigned to each group for each observed time point.

### Bioinformatics analysis of age-related renal transcriptome data.

Age-related renal cortical transcriptome data are publicly available from www.Nephroseq.org based on data sets derived from the Rodwell Aging Kidney (29) study and were analyzed. After excluding subjects with abnormal serum creatinine levels or blood pressure, or other comorbid conditions, such as diabetes and hypertension, the mRNA expression levels of GSK3β were retrieved and analyzed. To further investigate the biological pathways associated with GSK3β in normal and diseased glomeruli, glomerular transcriptome data originating from the Ju CKD Glom study (30) were analyzed by GSEA using the expert-curated kidney-aging-related gene set for specimens with high expression of GSK3β versus those with low expression of GSK3β based on gene expression microarray analysis. GSEA was performed by employing GSEA v4.1.0 software (http://software.broadinstitute.org/gsea/index.jsp). In addition, GPS 5.0 (http://gps.biocuckoo.cn/) was used to predict GSK3β phosphorylation consensus motifs in the amino acid sequences of p16^INK4A^ (NCBI accession number AAK83159.1) and p53 (NCBI accession number BAA82343.1).

### Urinary and serum measurements.

Equal volumes of urine samples were examined by SDS-PAGE followed by Coomassie Brilliant Blue (Sigma-Aldrich) staining. Urine albumin concentrations were assayed using a mouse albumin ELISA quantitation kit (Bethyl Laboratories Inc). Urine and serum creatinine levels were measured by a creatinine assay kit (BioAssay Systems) according to the manufacturer’s instructions. Albumin-to-creatinine ratios were calculated to assess the severity of albuminuria.

### Glomerular isolation and primary culture of podocytes.

Glomerular isolation from mouse kidneys was carried out as reported previously (21). Briefly, mice were euthanized and immediately perfused via left ventricular cannulation with ice-cold, sterile phosphate-buffered saline (PBS) until the kidneys had blanched. After the left kidney was resected for histological and other examinations, the right kidney was further perfused via abdominal aorta cannulation with PBS containing Dynabeads M-450 (Dynal Biotech ASA) or magnetic iron oxide particles (Sigma-Aldrich). Then, the right kidney was collected and cortices were minced into 1-mm^3^ pieces and digested in collagenase A at 37°C for 30 minutes with gentle shaking. Tissues were pressed gently through a 100-μm cell strainer (BD Falcon), and glomeruli were gathered using a magnetic particle concentrator. Primary podocytes were prepared from the isolated glomeruli as described previously (21). In brief, enriched glomeruli were plated on collagen type I–coated Petri dishes at 37°C in RPMI 1640 medium (Life Technologies) supplemented with 10% fetal bovine serum (FBS, Life Technologies), 1 mM sodium pyruvate (Sigma-Aldrich), 100 μg/mL streptomycin, and 100 U/mL penicillin (Life Technologies) in a humidified incubator with 5% CO_2_. Podocytes at passage 1 or 2 were characterized by the expression of multiple podocyte-specific markers and used in subsequent experiments.

### Cell culture and transfection.

Conditionally immortalized mouse podocytes in culture were a gift from Stuart Shankland (University of Washington, Seattle, Washington, USA) and cultured under permissive conditions as described previously (49). Briefly, cells were cultured in RPMI 1640 medium containing 10% FBS, 0.075% sodium bicarbonate (Sigma-Aldrich), 1 mM sodium pyruvate, 100 U/mL penicillin, and 100 μg/mL streptomycin in a humidified incubator with 5% CO_2_. Cells were cultured at 33°C with 50 U/mL recombinant mouse IFN-γ (Millipore) on collagen type I–coated plastic Petri dishes and were transferred to a 37°C incubator without IFN-γ to induce differentiation. Conditionally immortalized podocytes were transfected by using Lipofectamine 3000 reagent (Invitrogen) according to the manufacturer’s instructions. Transient transfection of primary podocytes was conducted via electroporation by using an Amaxa Nucleofection kit (Lonza Bioscience) as previously described (60). The transfection efficiency was evaluated based on HA expression.

### SA-β-gal activity staining.

Senescent cells are characterized by the overexpression and accumulation of endogenous lysosomal β-galactosidase, a hydrolase that catalyzes the hydrolysis of β-galactosides into monosaccharides only in senescent cells (31). For detection of SA-β-gal activity, cryosections of mouse kidneys or cultured podocytes were processed by using a commercial kit (9860, Cell Signaling Technology). The frozen sections were then counterstained with Nuclear Fast Red (N3020, Sigma-Aldrich).

### Flow cytometric cell cycle analysis.

Primary podocytes were harvested and fixed with 70% ethanol for 30 minutes at 4°C. Cells were centrifuged at 500*g*, washed with PBS, followed by ribonuclease (100 μg/mL) treatment for 10 minutes at 37°C and propidium iodine (50 μg/mL) labeling. Cell cycle analysis was performed with a FACSCalibur flow cytometer (Becton Dickinson) at an excitation wavelength of 488 nm, and data were analyzed by using ModFit LT software (Verity Software House).

### Western immunoblot analysis and immunoprecipitation.

Isolated renal glomeruli or whole kidney specimens were homogenized and cultured podocytes were lysed in radioimmunoprecipitation (RIPA) buffer supplemented with protease inhibitor cocktail (4693159001, Roche Diagnostics). Samples were processed for immunoblot analysis as specified previously (21) by using primary and secondary antibodies. Immunoprecipitation was performed as previously described (21), and immunoprecipitates were processed for immunoblot analysis for indicated proteins.

### Clinical studies.

For examination of age-related histologic features of the kidney, discarded non-neoplastic nephrectomy specimens were procured from patients who underwent radical nephrectomy due to renal tumor at different ages (young, less than 30 years old; middle-aged, 30–59 years old; older subjects, 60–79 years old) with exclusion of those with abnormal serum creatinine levels or blood pressure as well as comorbid conditions, such as diabetes, hypertension, or coexisting kidney diseases. Kidney specimens were prepared and banked at the Renal Pathology Laboratories at the First Affiliated Hospital of Zhengzhou University. Additionally, psychiatric patients receiving lithium carbonate treatment and psychiatric patients matched for age and sex but never treated with lithium carbonate were included in this study. All clinical and biochemical data were collected from medical chart reviews. Exclusion criteria included noncompliant patients, anecdotal evidence of drug overdose or toxicity, coexisting renal diseases as demonstrated by kidney dysfunction, abnormal urinalysis or imaging, and underlying conditions like diabetes, hypertension, and malignancy. Excess samples of clean-catch midstream urine were collected during the routine health checkups from all patients and prepared for analyses of urinary exfoliated cells.

### Immunofluorescent staining.

Frozen kidney sections or cultured podocytes were fixed with 4% paraformaldehyde (Sigma-Aldrich), permeabilized, and stained with primary antibodies followed by secondary antibody staining using Alexa Fluor 488 or 594. Filamentous actin (F-actin) in podocytes was stained by rhodamine-phalloidin (PHDR1, Cytoskeleton Inc.). Finally, sections or cells were counterstained with 4′,6-diamidino-2-phenylindole (DAPI) (ab104139, Abcam) and visualized using a fluorescence microscope (EVOS XL Core Imaging System, Thermo Fisher Scientific) or a Leica TCS SP5 laser scanning confocal microscope.

### Renal histology assessment and immunohistochemical analysis.

Formalin-fixed, paraffin-embedded kidney specimens were prepared as 3-μm-thick sections. For general histology, sections were processed for PAS and Masson trichrome staining by routine procedures. The morphologic features of all the sections were assessed by a single observer in a blinded manner. Global glomerulosclerosis was quantified by counting the number of globally sclerotic glomeruli per total number of glomeruli per specimen. The score of interstitial fibrosis was estimated by semiquantitative morphometric analysis of the Masson trichrome staining that was graded as follows: 0, <5% of the interstitial area affected; 1, 5% to 25% of the interstitial area affected; 2, >25% to 50% of the interstitial area affected; and 3, >50% of the interstitial area affected. Peroxidase immunohistochemical staining was conducted by using Vectastain ABC kits (Vector Laboratories) and primary antibodies against indicated proteins. As negative controls, the primary antibody was replaced with IgG isotype controls from the same species and no staining was noted. Computerized morphometry of immunohistochemical staining was performed as described previously (61) by using Image-Pro Plus software (Media Cybernetics).

### Transmission electron microscopy.

Kidney cortical tissues were cut into small pieces (1 mm^3^), fixed with 2.5% glutaraldehyde, and embedded in Epon 812 (Electron Microscopy Sciences). Samples were evaluated by an investigator in a blinded manner. The length of the peripheral GBM was measured by using ImageJ software (NIH) and the number of slit pores overlying this GBM length was counted. The arithmetic mean of the foot process width was calculated by using an equation as described previously (62).

### Analyses of urinary exfoliated cells.

Urinary exfoliated cells were isolated using a previously described protocol (43) with minimal modification. In brief, urine samples were centrifuged and urine sediments were deposited to a slide evenly by using a cell spreader. Cells were fixed with 4% paraformaldehyde, permeabilized, and stained sequentially with primary antibodies and Alexa Fluor 350, 488, or 594 secondary antibodies, followed by nuclear counterstaining with DAPI. The results were visualized using a fluorescence microscope.

### Statistics.

All in vitro experiments were performed at least 3 times. Numerical data are presented as mean ± SD. Statistical analysis was performed using GraphPad Prism 8.0. Comparisons among multiple groups were performed using 1-way ANOVA alone or followed by Tukey’s test. Data from 2 groups were compared by 2-tailed, unpaired Student’s *t* test. Linear regression analysis was applied to test possible relationships between 2 parameters. Categorical data were analyzed using Fisher’s exact test. Matched categorical data of the staining patterns of urinary cells were analyzed by Mantel-Haenszel χ^2^ test. *P* < 0.05 was considered statistically significant.

### Study approval.

All animal experiments were conducted according to protocols approved by the Institutional Animal Care and Use Committee (IACUC) at the Rhode Island Hospital and the University of Toledo. The clinical study of urine samples and discarded nephrectomy specimens was approved by the Institutional Review Board of the First Affiliated Hospital of Zhengzhou University in Zhengzhou, China, and conformed to the ethical guidelines of the 1975 Declaration of Helsinki.

## Author contributions

RG devised the conceptual ideas. YF and BC performed the in vitro and animal experiments. BC and YG contributed to animal breeding. WTG contributed to histologic interpretation. YF and ZL performed the clinical confirmatory study. YF, BC, LDD, and RG analyzed data. DM, AFG, and LDD contributed to discussion. YF, AYG, and RG wrote the manuscript. LDD contributed to manuscript revision. All authors agreed that the entire concept and ownership of this work belong to RG. All authors approved the final version of the manuscript.

## Supplementary Material

Supplemental data

## Figures and Tables

**Table 1:**
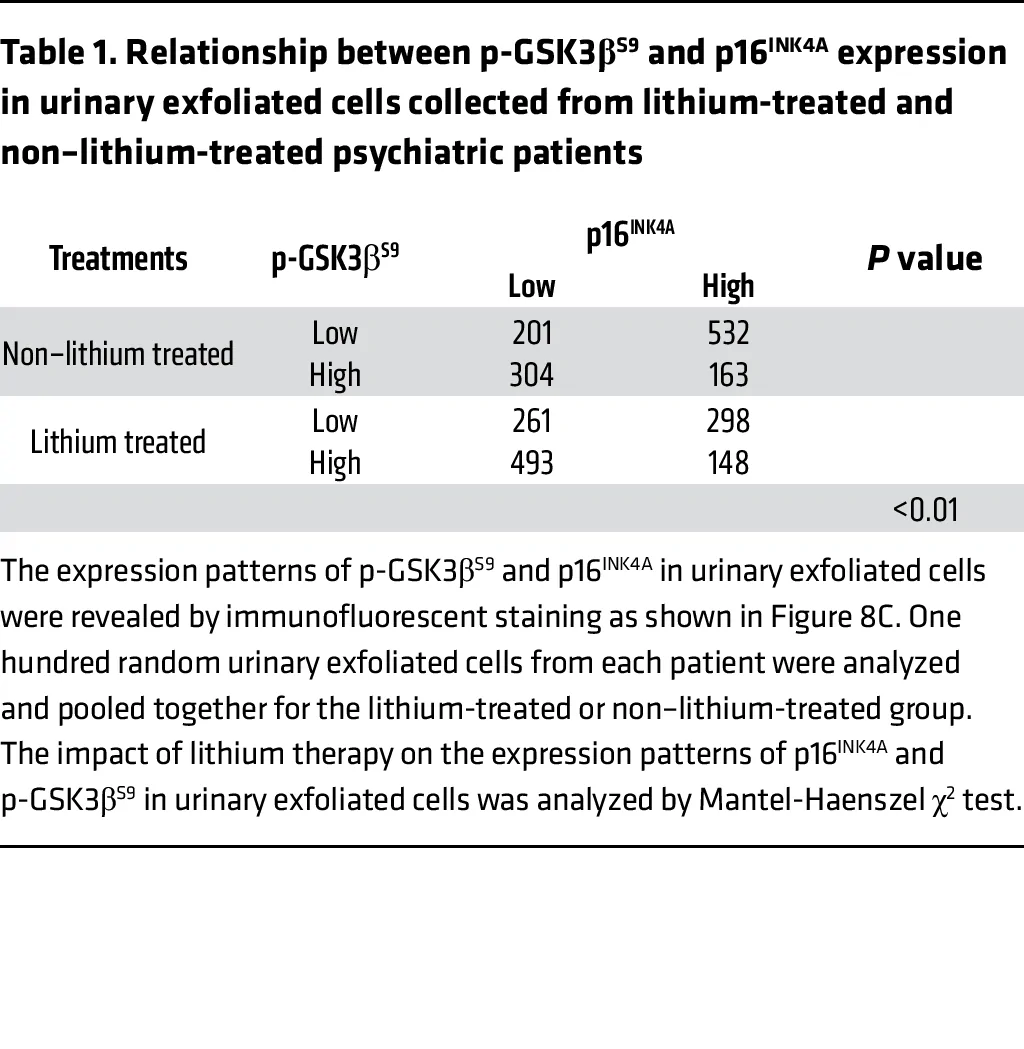
Relationship between p-GSK3β^S9^ and p16^INK4A^ expression in urinary exfoliated cells collected from lithium-treated and non–lithium-treated psychiatric patients

## Version 1 02/15/2022

Electronic publication

## Version 2 08/31/2026

Corrected an error in Supplemental Figure 5A in the WT-1/Ki67/DAPI Con image
