# Corrigendum to Age-related GSK3β overexpression drives podocyte senescence and glomerular aging

**DOI:** 10.1172/JCI210927

**Published:** 2026-09-01

**Authors:** Yudong Fang, Bohan Chen, Zhangsuo Liu, Athena Y. Gong, William T. Gunning, Yan Ge, Deepak Malhotra, Amira F. Gohara, Lance D. Dworkin, Rujun Gong

Original citation: *J Clin Invest*. 2022;132(4):e141848. https://doi.org/10.1172/JCI141848

Citation for this corrigendum: *J Clin Invest*. 2026;136(17):e210927. https://doi.org/10.1172/JCI210927

The authors recently became aware of an error in Supplemental Figure 5A in the WT-1/Ki67/DAPI Con image. The corrected figure, based on the original source data, is provided in the updated supplemental materials.

The authors regret the error.

## Version 1 09/01/2026

Electronic publication

