# Supplementary material for: Age-related GSK3β overexpression drives podocyte senescence and glomerular aging
Source: J Clin Invest. 2022 Feb 15;132(4):e141848. doi: 10.1172/JCI141848 (PMC8843754; doi:10.1172/JCI141848)
Supplement: Supplementary file 2 [file jci-132-141848-s010.pdf]

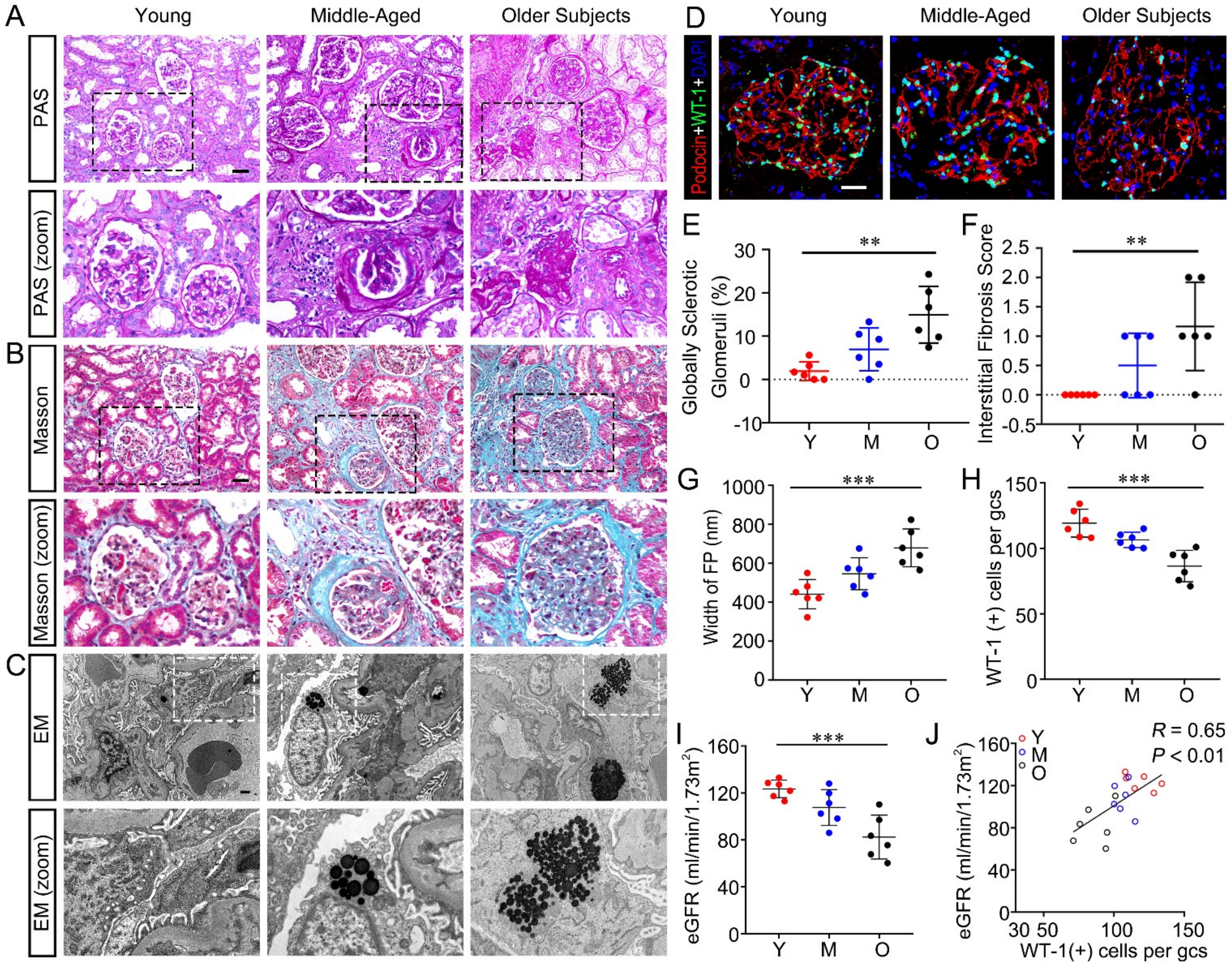

Supplemental Figure 1. Age-related changes of the kidney in men are associated with histological features of podocyte senescence, degeneration and loss. Non-neoplastic nephrectomy specimens were procured from patients who underwent radical nephrectomy due to renal tumor at different ages (Young or Y, <30 years old; Middle-aged or M, 30 to 59 years old; Older subjects or O, 60 to 79 years old) with exclusion of those with abnormal serum creatinine levels or blood pressure as well as comorbid conditions, such as diabetes, hypertension or coexisting kidney diseases. (A-D) Tissues were processed for (A) PAS staining (Scale bar=50μm), (B) Masson trichrome staining (Scale bar=50μm), (C) transmission electron microscopy (EM; Scale bar=1μm) with zoomed-in views of boxed regions, and (D) fluorescent immunohistochemistry staining for WT-1 and podocin followed by counterstaining with 4′,6-diamidino-2-phenylindole (DAPI; Scale bar=20μm). (E) The percentage of glomeruli with global glomerulosclerosis. ***P*<0.01 (n=6). (F) Semi-quantitative scoring of renal interstitial fibrosis based on Masson trichrome staining. ***P*<0.01 (n=6). (G) Quantification of the width of podocyte foot processes (FP) based on EM. ****P*<0.001 (n=6). (H) The average number of WT-1 positive cells per glomerular cross section (gcs) per patient. ****P*<0.001 (n=6). (I) Estimated glomerular filtration rates (eGFR) diminished with aging. ****P*<0.001 (n=6). (J) Linear regression analysis reveals that the average number of WT-1 positive cell per gcs per patient correlated with eGFR in different age groups (n=6). The Spearman correlation coefficient (*R*) and *P* values are shown. Data are expressed as mean±SD. Panels E-I were analyzed by one-way ANOVA followed by Tukey's test. Panel J was analyzed by linear regression analysis.

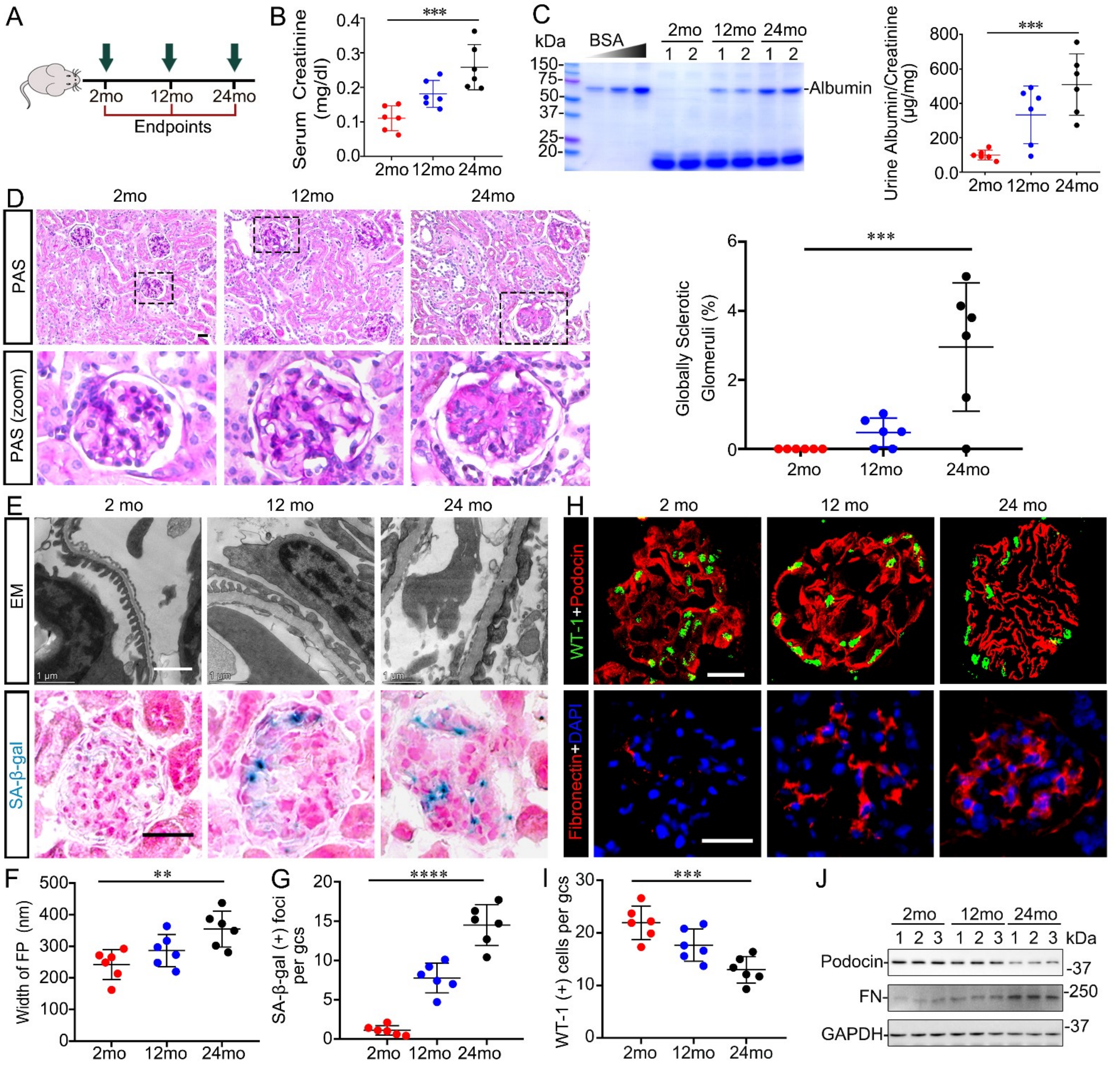

Supplemental Figure 2. Kidney aging is associated with podocyte senescence, degeneration and loss in mice. (A) Schematic diagram illustrates the animal experimental design. (B) Serum creatinine levels modestly but significantly increased with age. ****P*<0.001 (n=6). (C) Spot urine was collected at the indicated time points, and an aliquot (20 µl) was subjected to SDS-PAGE followed by Coomassie brilliant blue staining. Bovine serum albumin (BSA; 1, 2, and 4 µg) served as standard controls. Spot urine samples were subjected to albumin ELISA analysis with adjustment for urine creatinine concentrations. ****P*<0.001 (n=6). (D) Representative images show PAS staining with zoomed-in views of boxed regions. Scale bar=20µm. The percentage of glomeruli with global glomerulosclerosis progressively increased with age. ****P*<0.001 (n=6). (E) Kidney specimens were subjected to electron microscopy (EM; Scale bar=1µm) analysis and the senescence-associated β-galactosidase activity (SA-β-gal) staining (Scale bar=20µm). (F) Quantification of podocyte foot process (FP) width based on EM. ***P*<0.01 (n=6). (G) Absolute count of the average number of SA-β-gal-positive foci per glomerular cross section (gcs) per mouse. *****P*<0.0001 (n=6). (H) Kidney specimens were subjected to immunofluorescence staining for WT-1 and podocin, or for fibronectin followed by counterstaining with 4′,6-diamidino-2-phenylindole (DAPI; Scale bar=20µm). (I) Absolute count of the average number of WT-1-positive podocytes per gcs per mouse. ****P*<0.001 (n = 6). (J) Representative immunoblot analysis of isolated glomeruli for podocin and fibronectin (FN). GAPDH served as a loading control. Data are expressed as mean±SD. Panels B-D, F, G and I were analyzed by one-way ANOVA.

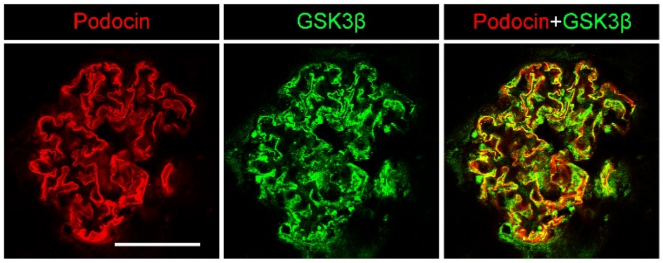

Supplemental Figure 3. Glomerular expression of GSK3β is mostly located to podocytes in aging mice. Kidney specimens were collected from wild-type mice aged 12 months as elaborated in Figure 2 and were processed for dual-color fluorescent immunohistochemistry staining for GSK3β and the podocyte maker podocin. Scale bar=50µm.

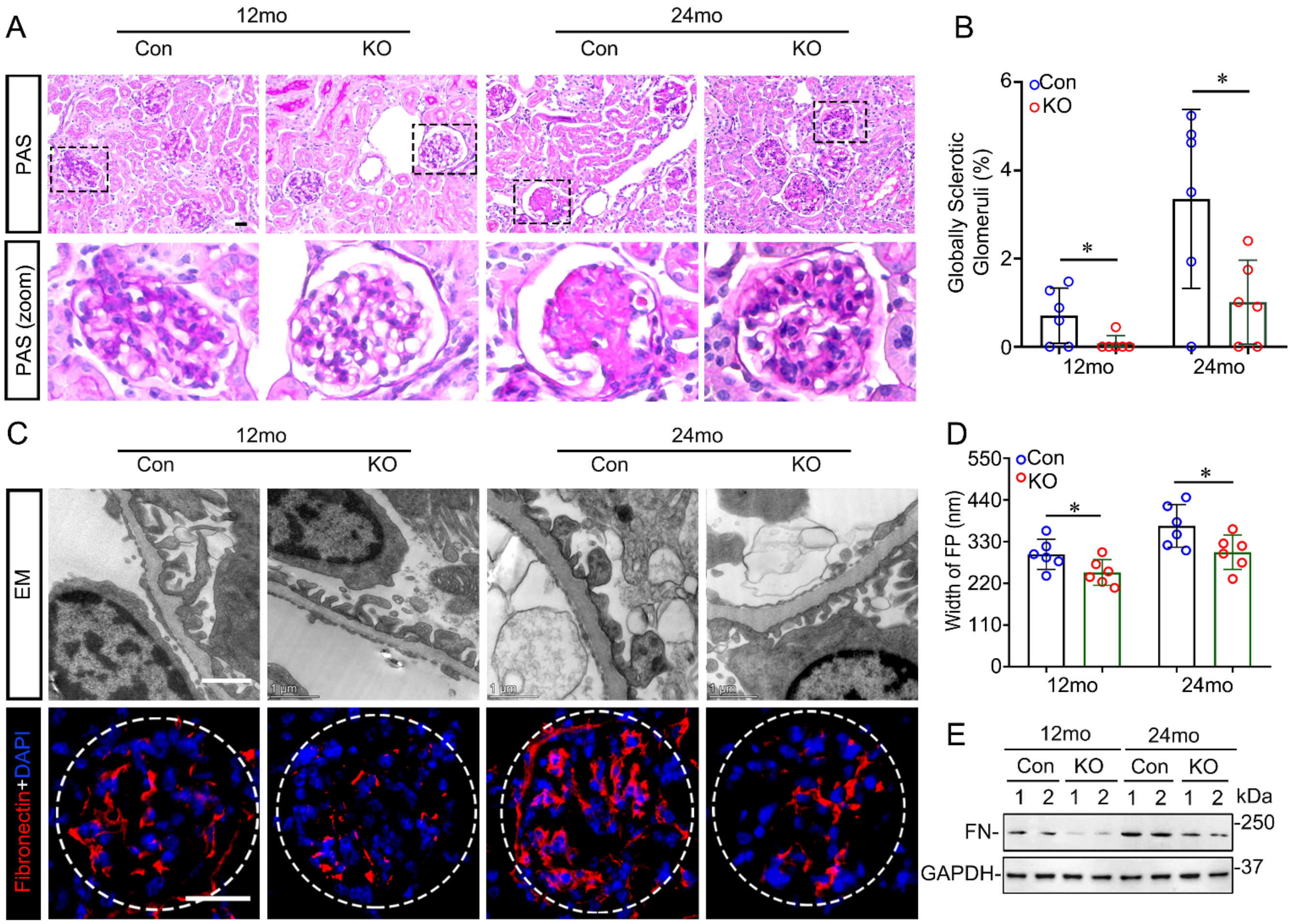

Supplemental Figure 4. Podocyte-specific ablation of GSK3β in mice mitigates podocyte senescence and kidney aging. (A) Representative micrographs with zoomed-in views of boxed regions demonstrate PAS staining of kidneys from control mice (Con) and mice with podocyte-specific GSK3β knockout (KO) at 12 or 24 months of age. Scale bar=20μm. (B) The percentage of glomeruli with global glomerulosclerosis was decreased significantly in KO mice as compared with Con mice at 12 or 24 months old. *P<0.05 (n=6). (C) Transmission electron microscopy (EM) of age-related ultrastructural lesions of podocytes in kidney tissues procured from Con and KO mice (scale bar=1μm). Kidney specimens procured from each group were also prepared for immunofluorescence staining for fibronectin followed by 4′,6-diamidino-2-phenylindole (DAPI) counterstaining. Scale bar=20μm. (D) Quantification of podocyte foot process (FP) width based on EM. *P<0.05 (n=6). (E) Representative immunoblot analysis of isolated glomeruli for fibronectin (FN). GAPDH served as a loading control. Data are expressed as mean±SD. Panels B and D were analyzed by 2-tailed unpaired Student’s t test.

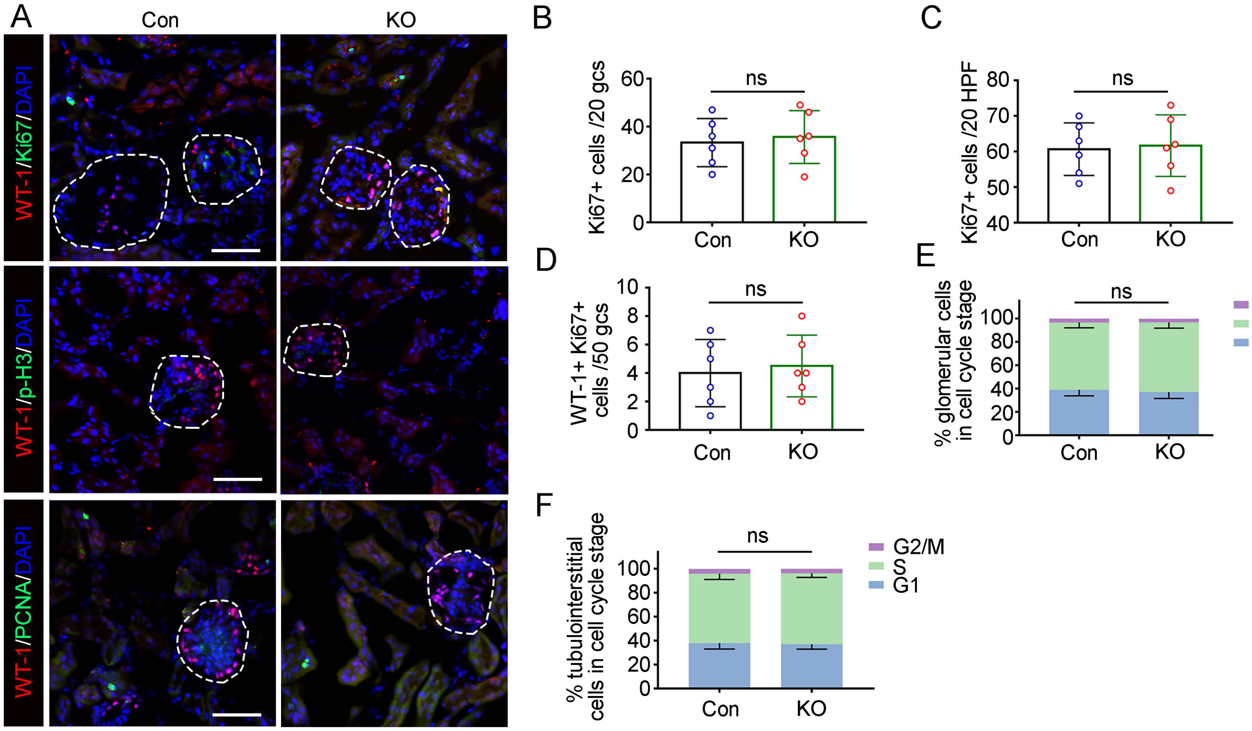

Supplemental Figure 5. Podocyte-specific ablation of GSK3β barely affects renal cell cycle profiles during kidney aging. (A) Mice were treated as elaborated in Figure 3A. Kidney specimens collected from Control (Con) and KO mice at 24 months old were processed for fluorescent immunohistochemistry staining for Ki67, proliferating cell nuclear antigen (PCNA), phospho-histone H3 (p-H3) and WT-1, followed by 4′,6-diamidino-2-phenylindole (DAPI) counterstaining, as shown by representative micrographs. Scale bar=50µm; (B-D) Quantification of the amount of Ki67 positive (Ki67+) cells (B) per 20 glomerular cross sections (gcs) as outlined with dashed lines in (A) or (C) per 20 high-power fields (HPF) of the renal tubulointerstitium, or (D) glomerular cells positive for both Ki67 and WT-1 staining per 50 gcs by absolute counting (n=6). (E-F) Cell cycle distribution of the proliferative cells in (E) glomeruli or (F) renal tubulointerstitium. ns, not significant (n=6). Data are expressed as mean±SD. Panels B-F were analyzed by 2-tailed unpaired Student's *t* test.

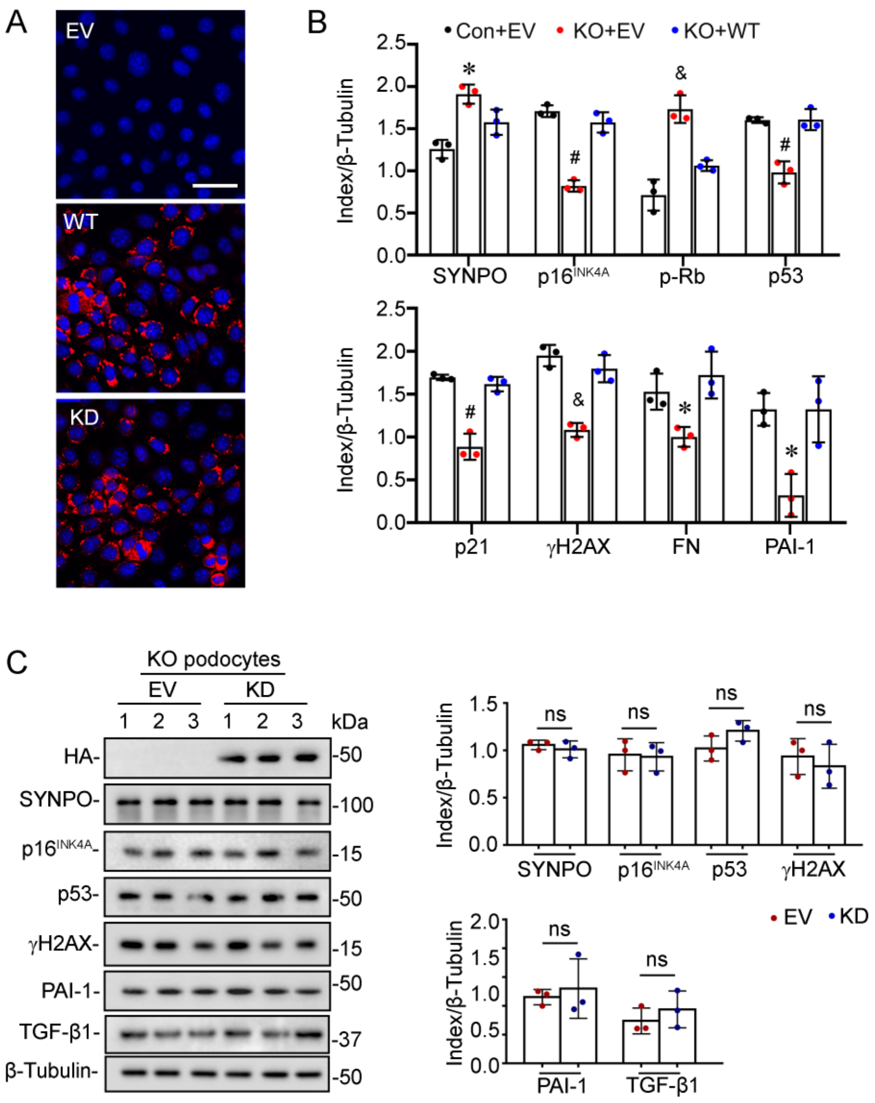

Supplemental Figure 6. Reconstitution of the kinase-dead mutant of GSK3β in KO podocytes barely affects cellular senescence and senescence-associated secretory phenotypes (SASP). (A) Primary podocytes were prepared from Control (Con) mice and mice with podocyte-specific GSK3β knockout (KO) as elaborated in Figure 4, and processed for electroporation-based transient transfection with either the empty plasmid vector (EV) or plasmids encoding the hemagglutinin (HA)-conjugated wild-type (WT) GSK3β or kinase-dead mutant (KD) of GSK3β by using the Amaxa Nucleofection kit. Cells were fixed and subjected to HA staining (red) with 4′,6-diamidino-2-phenylindole counterstaining (blue), which revealed a satisfactory transfection efficiency of approximately 70%. Scale bar=50µm; (B) Densitometric analysis of the expression of indicated molecules as relative levels normalized to β-Tubulin based on immunoblot analysis corresponding to Figure 4C. $^{*}P<0.05$, $^{\&}P<0.01$, $^{\#}P<0.001$ *versus* other groups (n=3). (C) After different treatments, KO cells were processed for immunoblot analysis for indicated molecules followed by densitometric analysis. ns, not significant (n=3). Data are expressed as mean±SD. Panel B was analyzed by one-way ANOVA followed by Tukey's test. Panel C was analyzed by 2-tailed unpaired Student's *t* test.

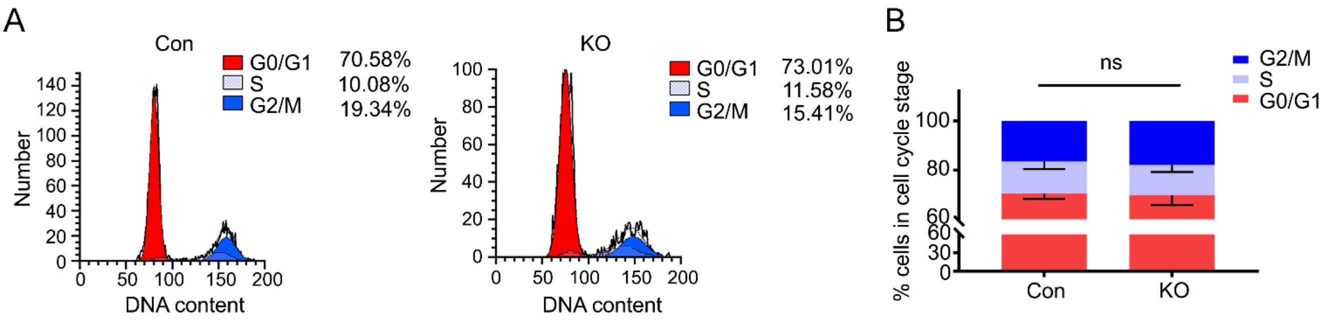

Supplemental Figure 7. Loss of GSK3β has no noticeable effect on podocyte cell cycle profiles. (A) Primary podocytes were prepared from control mice (Con) and mice with podocyte-specific GSK3β knockout (KO) as elaborated in Figure 4 and cultured. Cells were then collected, fixed and stained with propidium iodide, followed by cell cycle analysis by flow cytometry-based DNA content measurement. Representative flow cytometry results are shown. (B) The proportion of cells in G0/G1, S and G2/M phases in different groups. ns, not significant (n=3). Data are expressed as mean±SD and statistically analyzed by 2-tailed unpaired Student's *t* test.

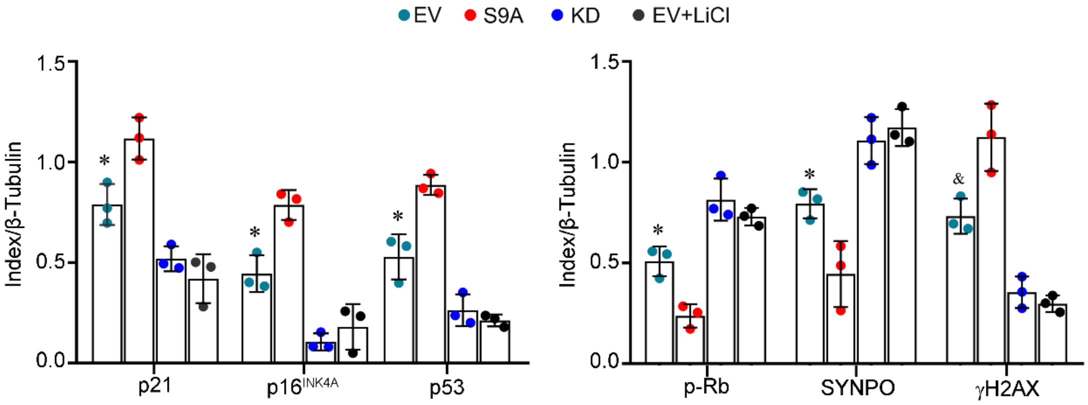

Supplemental Figure 8. Senescence signaling in podocytes is promoted by forced expression of S9A but mitigated by lithium or ectopic expression of KD. Conditionally immortalized murine podocytes were treated as elaborated in Figure 6 and processed for immunoblot analysis as shown by a representative blot in Figure 6C. Densitometric analysis of the expression of indicated molecules as relative levels normalized to β-Tubulin based on immunoblot analysis. $^{*}P<0.05$, $^{\&}P<0.01$ *versus* other groups (n=3). Data are expressed as mean±SD and statistically analyzed by one-way ANOVA followed by Tukey's test.

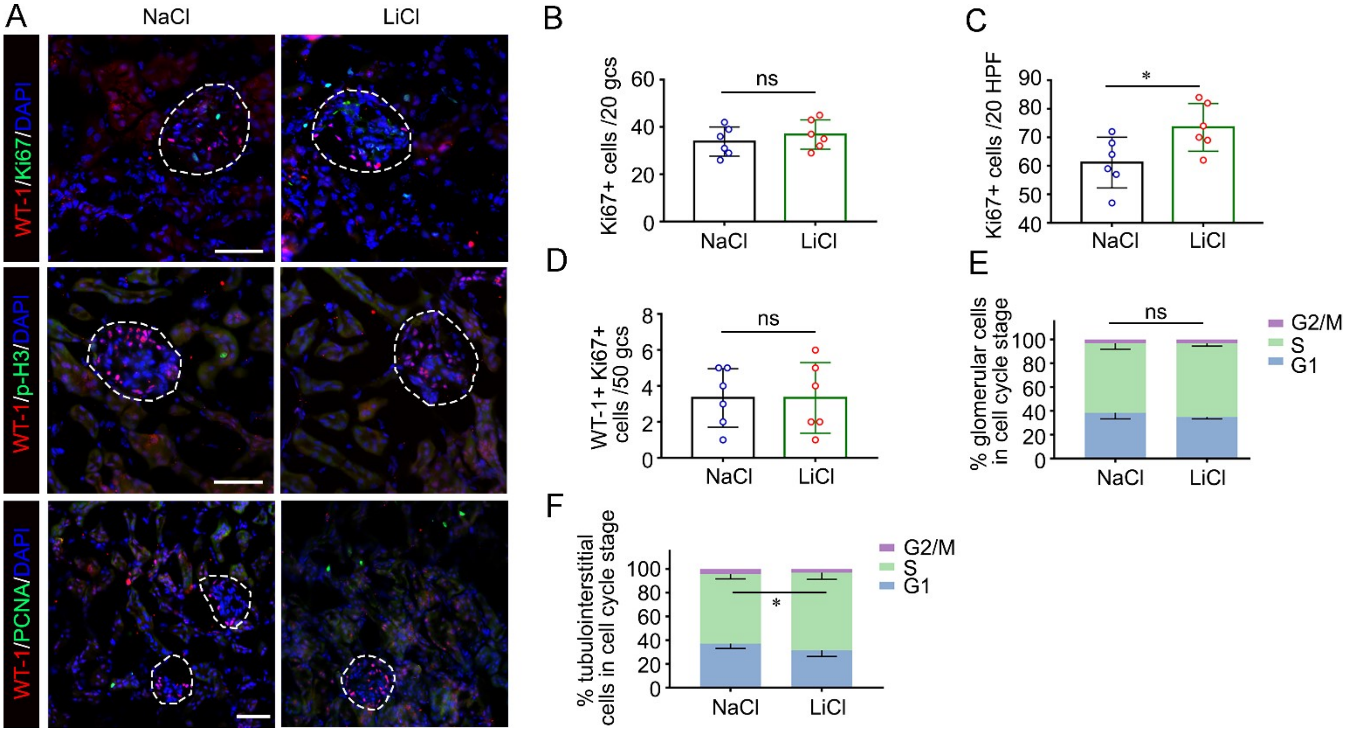

Supplemental Figure 9. Microdose lithium treatment minimally affects renal cell cycle profiles during kidney aging. (A) Mice were treated as elaborated in Figure 7C. Kidney specimens collected from lithium chloride- or sodium chloride-treated mice at 18 months old were processed for fluorescent immunohistochemistry staining for Ki67, PCNA, p-H3 and WT-1, followed by 4′,6-diamidino-2-phenylindole (DAPI) counterstaining. Representative micrographs are shown. Scale bar=50µm; (B-D) Quantification of the amount of Ki67 positive (Ki67+) cells (B) per 20 glomerular cross sections (gcs) as outlined with dashed lines in (A) or (C) per 20 high-power fields (HPF) of the renal tubulointerstitium, or (D) glomerular cells positive for both Ki67 and WT-1 staining per 50 gcs by absolute counting (n=6). (E-F) Cell cycle distribution of the proliferative cells in glomeruli (E) or renal tubulointerstitia (F) cells. **P*<0.05; ns, not significant (n=6). Data are expressed as mean±SD and analyzed by 2-tailed unpaired Student’s *t* test.

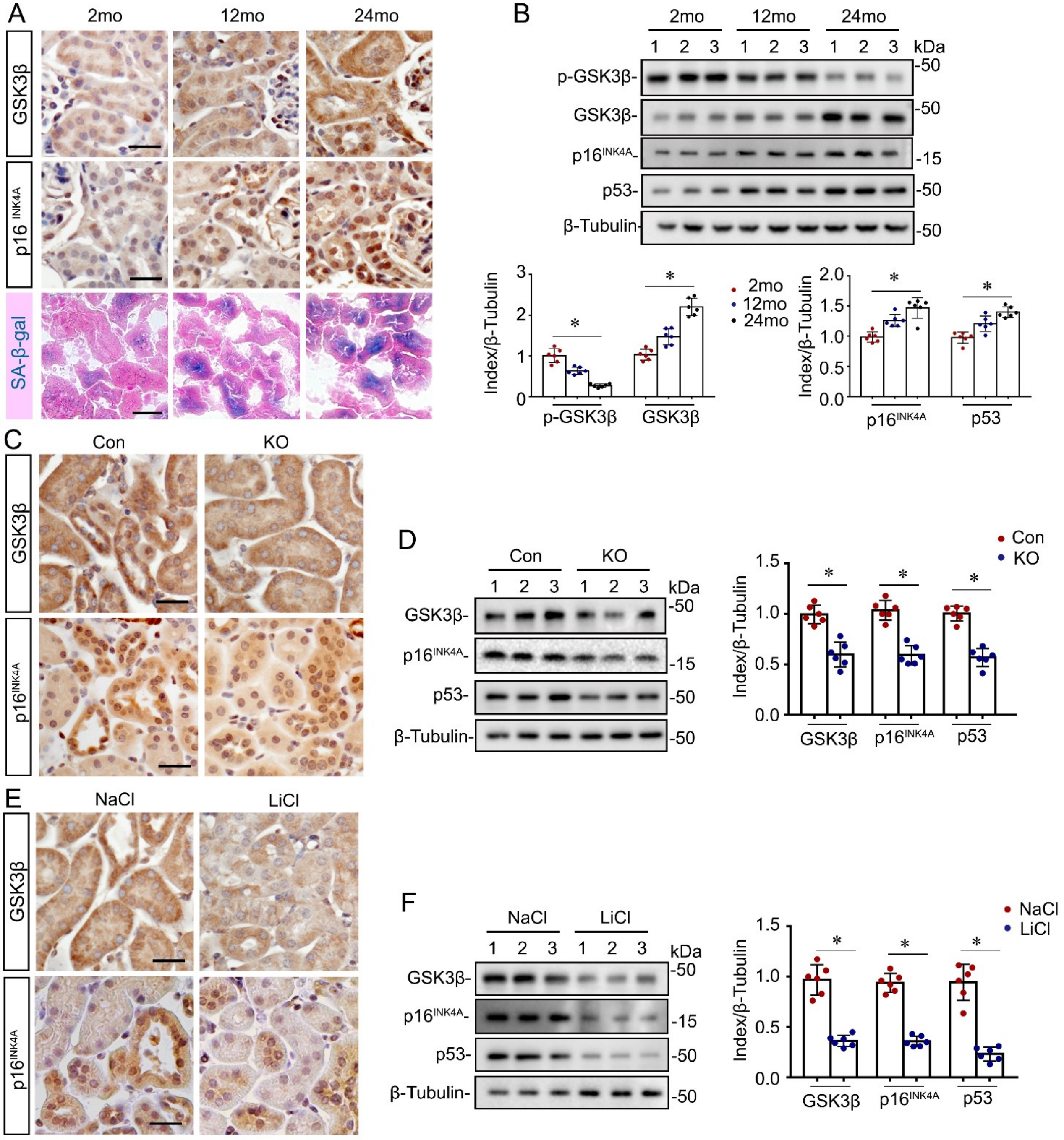

Supplemental Figure 10. Age-related overexpression of GSK3β in renal cortical tubules is associated with kidney aging and could be targeted by microdose lithium. (A) Kidney specimens were collected from wild-type mice at 2, 12 or 24 months old as elaborated in Supplemental Figure 2A, and processed for immunohistochemistry staining for GSK3β or p16^{INK4A}, or for SA-β-gal activity staining. Scale bar=25μm; (B) Representative immunoblot analysis of whole kidney homogenates. Densitometric analyses of the expression levels of indicated proteins, presented as relative levels normalized to β-tubulin based on immunoblot analysis. **P*<0.01 (n=6); (C) KO and control (Con) mice were treated as elaborated in Figure 3A. Kidney specimens collected at 24 months old were processed for immunohistochemistry staining for GSK3β or p16^{INK4A}. Scale bar=25μm; (D) Representative immunoblot analysis of whole kidney homogenates prepared at 24 months old. Densitometric analyses of the expression levels of indicated proteins, presented as relative levels normalized to β-tubulin based on immunoblot analysis; **P*<0.05 (n=6); (E) Mice were treated as elaborated in Figure 7C. Kidney specimens collected from lithium chloride- or sodium chloride-treated mice at 18 months old were processed for immunohistochemistry staining for GSK3β or p16^{INK4A}. Scale bar=25μm; (F) Representative immunoblot analysis of whole kidney homogenates prepared at 18 months old. Densitometric analyses of the expression levels of indicated proteins, presented as relative levels normalized to β-tubulin based on immunoblot analysis; **P*<0.01 (n=6). Data are expressed as mean±SD. Panel B was analyzed by one-way ANOVA. Panels D and F were analyzed by 2-tailed unpaired Student's *t* test.

Supplemental Table 1. Clinical and biochemical characteristics of psychiatric patients treated with lithium chloride and those without lithium chloride therapy.

	**Non-lithium treated** (n=12)	**Lithium treated** (n=12)	***P* value**
Demographics			
Age (years)	45.25±9.753	43.92±10.75	0.753
Sex (male/female)	7/5	5/7	0.684
Age at disease onset (years)	25.58±6.748	23.33±10.84	0.548
Course of disease (years)	19.67±5.158	20.58±5.418	0.675
BMI (kg/m^2)	23.22±2.049	22.31±2.682	0.359
Hypertension (yes/no)	0/12	0/12	NA
Diabetes (yes/no)	0/12	0/12	NA
Kidney diseases (yes/no)	0/12	0/12	NA
Laboratory data			
uACR (mg/mmol)	1.773±0.8999	1.113±0.5242	0.039
eGFR (CKD-EPI) (ml/min/1.73m^2)	101.00±8.622	109.10±8.917	0.033
Serum albumin (g/L)	43.91±3.060	44.07±3.043	0.900
Serum cholesterol (mmol/L)	4.397±1.341	3.923±1.088	0.353
Serum triglyceride (mmol/L)	1.999±1.353	1.549±1.079	0.378

Abbreviations: BMI, body mass index; eGFR, estimated glomerular filtration rate; NA, not applicable; uACR, urinary albumin/ creatinine.
